# Environmental effectiveness of the National Action Plan to Contain Antimicrobial Resistance: evidence from Chinese soil

**DOI:** 10.1093/nsr/nwag387

**Published:** 2026-06-24

**Authors:** Yuxiang Zhao, Zishu Liu, Xi Chen, Yue Huang, Shuxian Li, Xuemei Mao, Xiawan Zheng, Xiangwu Yao, Baolan Hu, Lizhong Zhu, Tong Zhang

**Affiliations:** Environmental Microbiome Engineering and Biotechnology Laboratory, Center for Environmental Engineering Research, Department of Civil Engineering, The University of Hong Kong, Hong Kong 999077, China; State Key Laboratory of Soil Pollution Control and Safety, Zhejiang University, Hangzhou 310058, China; College of Environmental and Resource Sciences, Zhejiang University, Hangzhou 310058, China; State Key Laboratory of Soil Pollution Control and Safety, Zhejiang University, Hangzhou 310058, China; College of Environmental and Resource Sciences, Zhejiang University, Hangzhou 310058, China; College of Natural Resources and Environment, Northwest A&F University, Yangling 712100, China; Environmental Microbiome Engineering and Biotechnology Laboratory, Center for Environmental Engineering Research, Department of Civil Engineering, The University of Hong Kong, Hong Kong 999077, China; Environmental Microbiome Engineering and Biotechnology Laboratory, Center for Environmental Engineering Research, Department of Civil Engineering, The University of Hong Kong, Hong Kong 999077, China; Environmental Microbiome Engineering and Biotechnology Laboratory, Center for Environmental Engineering Research, Department of Civil Engineering, The University of Hong Kong, Hong Kong 999077, China; Environmental Microbiome Engineering and Biotechnology Laboratory, Center for Environmental Engineering Research, Department of Civil Engineering, The University of Hong Kong, Hong Kong 999077, China; Environmental Microbiome Engineering and Biotechnology Laboratory, Center for Environmental Engineering Research, Department of Civil Engineering, The University of Hong Kong, Hong Kong 999077, China; State Key Laboratory of Soil Pollution Control and Safety, Zhejiang University, Hangzhou 310058, China; State Key Laboratory of Soil Pollution Control and Safety, Zhejiang University, Hangzhou 310058, China; College of Environmental and Resource Sciences, Zhejiang University, Hangzhou 310058, China; Zhejiang Key Laboratory of Water Pollution Control and Water Ecological Health, Zhejiang University, Hangzhou 310058, China; State Key Laboratory of Soil Pollution Control and Safety, Zhejiang University, Hangzhou 310058, China; College of Environmental and Resource Sciences, Zhejiang University, Hangzhou 310058, China; Environmental Microbiome Engineering and Biotechnology Laboratory, Center for Environmental Engineering Research, Department of Civil Engineering, The University of Hong Kong, Hong Kong 999077, China; School of Public Health, The University of Hong Kong, Hong Kong 999077, China; Department of Environmental Science and Engineering, Macau University of Science and Technology, Macao 999078, China; The University of Hong Kong Shenzhen Institute of Research and Innovation, Shenzhen 518057, China; The State Key Laboratory of Marine Environmental Health (SKLMEH), City University of Hong Kong, Hong Kong 999077, China

**Keywords:** soil, antibiotic resistance genes, connectivity, China, co-selection

## Abstract

Soil antibiotic resistance genes (ARGs) represent an emerging planetary health threat. However, the environmental impacts of antimicrobial resistance (AMR) control policies remain unclear. Based on 2243 Chinese metagenomes, we generated a 14-year (2009–2022) spatiotemporal profile of Chinese soil ARGs and developed an open-access platform based on the interactive map. Relative to pre-2015 samples, the relative abundance of total ARGs (52.6%) and Rank I ARGs (77.0%) in croplands decreased markedly after 2016, coinciding with the national AMR control policy period (2016–2020). Comparing soil resistomes globally (2556 metagenomes) revealed homogenization in croplands, reflecting convergent ARG profiles under similar agricultural pressures across regions. This underscores the need for a shift from national to global intervention. Our findings highlight the importance of coordinated strategies that combined chemical pollution control with agricultural best practices to curb ARGs’ dissemination under the One Health framework.

## Teaser

Chinese soil resistomes showed pronounced declines during the National Action Plan to Contain Antimicrobial Resistance, suggesting that AMR interventions can generate measurable environmental benefits.

## INTRODUCTION

Antimicrobial resistance (AMR) has emerged as an escalating global health concern, potentially causing 8.22 million premature deaths annually by 2050 [1]. The 79th High-Level Meeting on AMR emphasized the crucial role of the environment within the One Health framework in addressing and controlling AMR [2], as antibiotic resistance genes (ARGs) can be transmitted to humans from the environment [3]. Although the efficacy of AMR control strategies in clinical settings has been evaluated, their potential impacts on environmental resistomes remain unclear.

Soil is an essential component of One Health [4], acting as both a source and a reservoir [5]. The widespread detection of Rank I ARGs (theoretically enriched in human-associated environments [6]) in soils reveals a significant connectivity between soil and human resistome [7]. This cross-habitat transmission suggests an intense relationship between humans and soil in the dissemination of AMR [8]. Thus, it is hypothesized that the implementation of AMR control strategies could have impacts on the soil resistome. Indeed, while global-scale studies provide valuable insights into the profile of ARGs’ relative abundance [9], risks [8], hosts [10] and driving forces [11], they may obscure region-specific trends and temporal variations, particularly due to differences in the timing of AMR control policy implementation across countries [12]. In contrast, regional-scale analyses allow for more sensitive detection of localized responses to policy interventions and enable the identification of potential links between soil and human antibiotic resistance under uniform regulatory frameworks. Overall, conducting focused studies in prioritized regions is essential for understanding regional soil antibiotic resistomes and

their response to the implementation of AMR control strategies.

Against the backdrop of escalating global AMR, China stands out as an intriguing case in this context. Controlling soil ARGs poses a significant challenge for China, largely due to the prevalence of smallholder farming systems [13], the high density of livestock production [14] and the intensive use of antibiotics. Over the past decade, nationwide antibiotic stewardship, including the National Action Plan to Contain Antimicrobial Resistance (hereinafter referred to as the ‘Plan’), has offered a large-scale experiment. This unique combination enables us to address a fundamental question: How effectively can national-scale interventions reduce soil ARGs? Preliminary evidence suggests that the human density of antimicrobial use in China decreased from 4.7 to 4.0 (∼15%) defined daily doses (DDDs) per 1000 inhabitants per day from 2015 to 2022 [15], and the veterinary antimicrobial consumption decreased from 41.8 to 32.5 (∼25%) kilotons from 2015 to 2021 [16]. However, whether this translates to soil ARG depletion remains untested.

Herein, we used the most comprehensive soil metagenomic datasets (2243) of China to characterize the resistome in Chinese soils and to assess the connectivity of ARGs between Chinese and global soils (excluding Chinese samples). Meanwhile, we identified the main driving factors of soil ARGs by analyzing 437 chemical pollutants in 110 samples collected from Chinese cropland, along with related environmental factors and other parameters. Finally, we developed an open-access platform based on the interactive map for the visualization of Chinese soil resistome from 2009 to 2022. We aimed to address three key questions: (i) What are the changes of Chinese soil resistome before and after the implementation of AMR Plan? (ii) What are the differences and similarities between Chinese and global soil ARG resistomes? (iii) Which factors contribute to the persistence of ARGs?

## RESULTS

### The distribution and profile of the Chinese soil antibiotic resistome

To gain comprehensive insight into Chinese soil antibiotic resistomes, a database of 2243 metagenomic datasets was compiled (Fig. S1 and Data S1). Some multidrug resistance genes are mutation-dependent, where resistance arises from specific genetic alterations [17], whereas others function as regulatory genes that do not directly confer resistance but may contribute to the development of a resistance phenotype [18]. To minimize mis-annotation under similarity-based approach, genes associated with multidrug efflux pumps were excluded. In addition, Rank I ARGs, selected based on gene mobility, host pathogenicity and human-associated enrichment, were analyzed as a specific subgroup, with their relative abundance used as an indicator of the potential risk of each sample [6] (Data S2). A total of 2043 ARGs subtypes and 108 Rank I ARGs subtypes were detected in Chinese soils (across all land use types). On average, the relative abundance of total ARGs in Chinese soils reached 0.16 copies/cell, and that of Rank I ARGs reached 2.3 copies/1000 cells, which were similar to those observed in wastewater treatment plants’ effluent [8].

**Figure 1.**
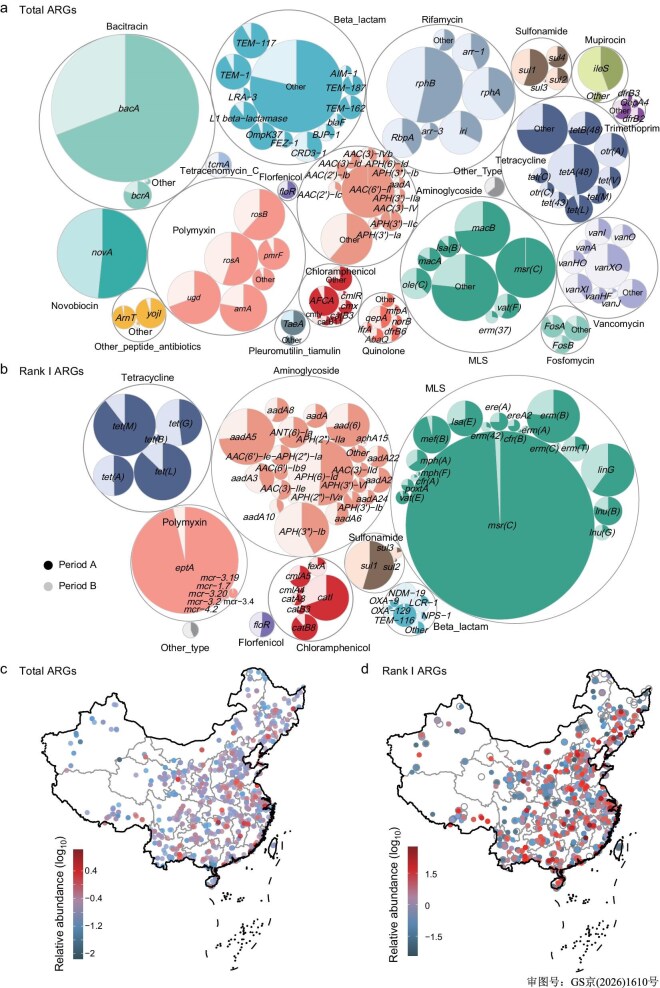
Composition and distribution of ARGs in Chinese soils. (a) The composition of total ARGs. The color of each circle represents the ARG type, and the square size indicates the proportion of each ARG subtype relative to its corresponding type and to the total relative abundance of all ARGs. Different colors represent different ARG types. The pie chart within each type’s circle indicates the distribution of subtypes in Period A and Period B, with darker colors indicating Period A and lighter colors indicating Period B. (b) The composition of Rank I ARGs. The color of each circle represents the Rank I ARG type, and the square size reflects the proportion of each Rank I ARG subtype relative to its corresponding type and to the total relative abundance of all Rank I ARGs. Rank I ARGs were determined based on ARG variants. Each ARGs subtype corresponds to the sum of these variants. Different colors represent different ARG types. The pie chart within each type’s circle indicates the distribution of subtypes in Period A and Period B, with darker colors indicating Period A and lighter colors indicating Period B. The ARGs variants are shown in Data S2. (c) The distribution of total ARGs. Log_10_ transformation was conducted for copies/cell. (d) The distribution of Rank I ARGs. The sum of Rank I ARGs was calculated based on the ARG variants. Log_10_ transformation was conducted for copies/1000 cells. The hollow circle represents the absence of Rank I ARGs.

To assess the potential impact of Plan (2016–2020) on soil resistomes, we compared the samples from two periods, i.e., 2015 or earlier (Period A, before the Plan) and 2016 or later (Period B, after the Plan), and applied the same temporal classification to the global dataset. The temporal trends of each subtype between Period A and Period B suggested that >70% of the subtypes exhibited a significant decline in Period B (Fig. 1a and b). Notably, several Rank I ARGs emerged exclusively in Period B, including *bla*_NDM-19_ and *mcr-1.7*.

We used three criteria to identify the core ARGs in soil [19], including overall abundance (being among the top 0.1% ARGs in mean relative abundance), ubiquity (detected in over 80% samples) and being frequently abundant (the relative abundance being in the top 80% in >50% of samples) (Data S3). Briefly, 15 subtypes (*bacA, novA, rphB, tetA(48)*, etc.) were identified as core ARGs in Chinese soils. Although these core ARGs subtypes accounted for only 0.73% of the total 2043 ARGs subtypes, their relative abundance reached 0.093 copies/cell, accounting for 58.8% of total ARGs. Aminoglycoside (35.2%), macrolide-lincosamide-streptogramin (MLS, 32.0%), tetracycline (9.6%), sulfonamide (5.9%) and beta-lactam (5.2%) were the most abundant Rank I ARGs types in Chinese soils (Fig. 1b). Finally, we created an interactive map as an open-access platform (https://smile.hku.hk/map/) to visualize the geographical distribution of ARGs and their relative abundance in Chinese soils (Fig. 1c and d).

### The antibiotic resistomes of different land use types

**Figure 2.**
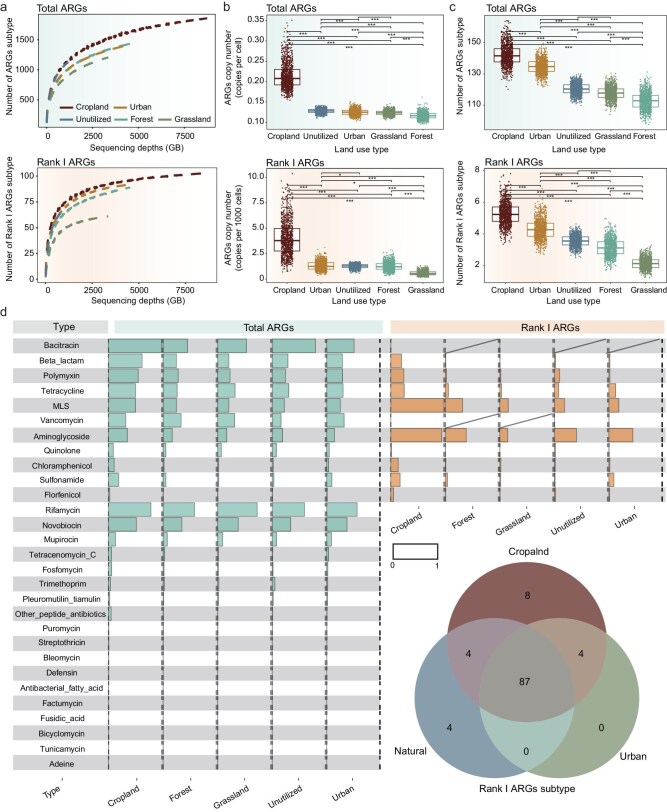
ARGs profiles across different land use types in Chinese soils. (a) ARGs rarefaction curves grouped by land use type. The rarefaction curves were calculated using total ARGs and Rank I ARGs subtype. (b) The normalized relative abundance of total ARGs and Rank I ARGs. A total of 999 rounds of sampling were performed with the same sample size for each land use type (unutilized land, 100 samples) in the calculation of both total ARGs and Rank I ARGs. (c) The normalized number of ARGs subtypes of total ARGs and Rank I ARGs. A total of 999 rounds of sampling were performed with the same sample size for each land use type (unutilized land, 100 samples) in the calculation of both total ARGs and Rank I ARGs. (d) The presence and relative abundance of total ARGs and Rank I ARGs types detected in different land use types. The relative abundance of total ARGs and Rank I ARGs was normalized separately. The subplot represents the subtypes of Rank I ARGs that were shared and unique to different land use types. Grassland, forest and unutilized land were combined into the natural group.

The Chinese soil metagenomic data were further divided into five groups based on land use type (cropland, urban, unutilized land, grassland and forest). The rarefaction curves of each land use type plateaued as shown in Fig. 2a. A total of 1857 ARGs subtypes (out of 2744 ARGs subtypes in the database) and 103 Rank I ARGs subtypes (out of 117 Rank I ARGs subtypes) were detected in Chinese cropland, 1.2 to 1.7 times higher than other land use types. Significant differences were also observed in the antibiotic resistomes between land use types, both in total ARGs and Rank I ARGs (Tables S1–S3). To ensure comparability across land use types, we randomly selected 100 samples per group and repeated this process 999 times. Results showed that cropland was the land use type with the highest relative abundance of total ARGs (0.21 copies/cell) and Rank I ARGs (4.0 copies/1000 cells), and the greatest ARGs diversity (total ARGs: 142.0 subtypes, Rank I ARGs: 5.3 subtypes) (Data S4), which were 1.1–6.9 times higher than other land use types (Fig. 2b and c). The unrarefied results are shown in Fig. S2 and were consistent with those obtained after rarefaction.

Bacitracin, rifamycin, beta-lactam, polymyxin and tetracycline were the most abundant ARGs types in cropland, accounting for 58.5%. MLS, aminoglycoside, tetracycline, polymyxin and beta-lactam were the most abundant Rank I ARGs types, accounting for 80.8%. Eight unique Rank I ARGs subtypes were observed in cropland, including *bla*_NDM-19_, *mcr-3.2, mcr-3.19 and mcr-3.20* (Fig. 2d). The Rank I ARGs for each group and their relative abundances are provided in Data S5. Considering their clinical significance, these ARGs warrant close monitoring.

### Comparative analysis of Rank I ARGs between Chinese and global cropland

**Figure 3.**
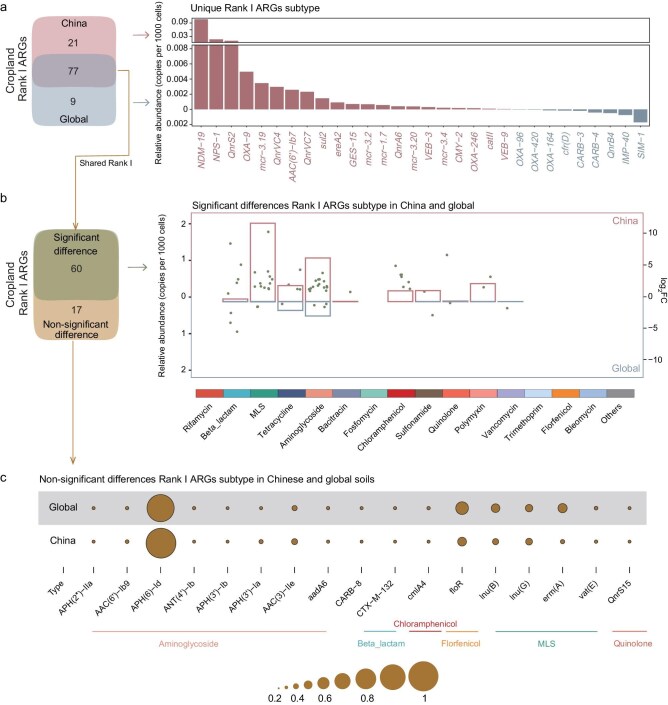
Differences in Rank I ARGs subtypes in cropland soils between China and global groups. The analysis included 2243 Chinese samples and 2556 samples from other countries, comprising the global group (2234 previously reported [8] and 322 newly introduced). Samples with a total relative abundance of Rank I ARGs equal to 0 were excluded from the analysis. (a) Unique Rank I ARGs subtypes in Chinese and global soils. The bar chart illustrates 21 unique Rank I ARGs in Chinese soil and 9 unique Rank I ARGs globally. The bar indicates the mean relative abundance in Chinese and global soils. Detailed values are shown in Data S15. (b) Shared Rank I ARGs subtypes with significant differences between Chinese and global soils (excluding Chinese samples). 60 Rank I ARGs that displayed significant differences between Chinese and global cropland soils are shown in the bar and node chart. Nodes represent Rank I ARGs showing significant differences between Chinese and global cropland soils and indicate the log_2_ fold change. The bar chart indicates the sum of types corresponding to Rank I ARGs subtypes that were significantly enriched in either Chinese or global soils. Detailed values are shown in Data S16. FC, fold change. (c) Shared Rank I ARGs subtypes with non-significant differences between Chinese and global soils. The relative abundance was normalized based on the maximum value.

To investigate the characteristics of ARGs in Chinese soils, we included 2556 previously reported global soil metagenomic datasets for analysis (for details, see section entitled ‘methods’; Data S6) [8]. The global dataset (excluding Chinese samples) was divided into high-income countries and low- and middle-income countries (LMICs). Results showed that the relative abundance of Rank I ARGs was significantly higher in LMICs than in high-income countries (*P* < 0.001), especially in cropland soils (Fig. S4). As cropland exhibited the highest risk and diversity of Rank I ARGs (Fig. 2), the differences in Rank I ARGs between Chinese and global cropland were further analyzed. A total of 107 Rank I ARGs subtypes (out of 117 Rank I ARGs subtypes) were identified in cropland soils across both China and other countries. Among these, 21 were exclusive to Chinese cropland soils, 9 were never found in Chinese cropland soils and 77 were shared (Fig. 3a and Data S7). The relative abundance of Rank I ARGs unique to Chinese croplands accounted for 2.9% of total Rank I ARGs, and included *bla*_NDM-19_, *mcr-3.4, mcr-3.20* and *mcr-1.7*. Meanwhile, Rank I ARGs never found in Chinese croplands accounted for 0.1% of total Rank I ARGs such as *CARB-4* and *IMP-40* (Fig. 3a).

Among the 77 shared Rank I ARGs subtypes, we further compared those that showed significant differences between Chinese and global cropland samples (Data S7). Of these, 48 subtypes were significantly abundant in Chinese samples (*P* < 0.05, accounting for 81.5%), while 12 subtypes were significantly higher in global samples (*P* < 0.05, accounting for 22.0%) (Fig. 3b). The remaining 17 Rank I ARGs subtypes showed no significant difference between Chinese and global cropland samples, with relative abundances of 11.0% and 22.8%, respectively. These subtypes were primarily associated with aminoglycoside, MLS, beta-lactam, florfenicol and tetracycline (Fig. 3c). Thus, these results indicated a distinct regional distribution pattern of Rank I ARGs in cropland.

### Temporal trends for Chinese and global ARGs

**Figure 4.**
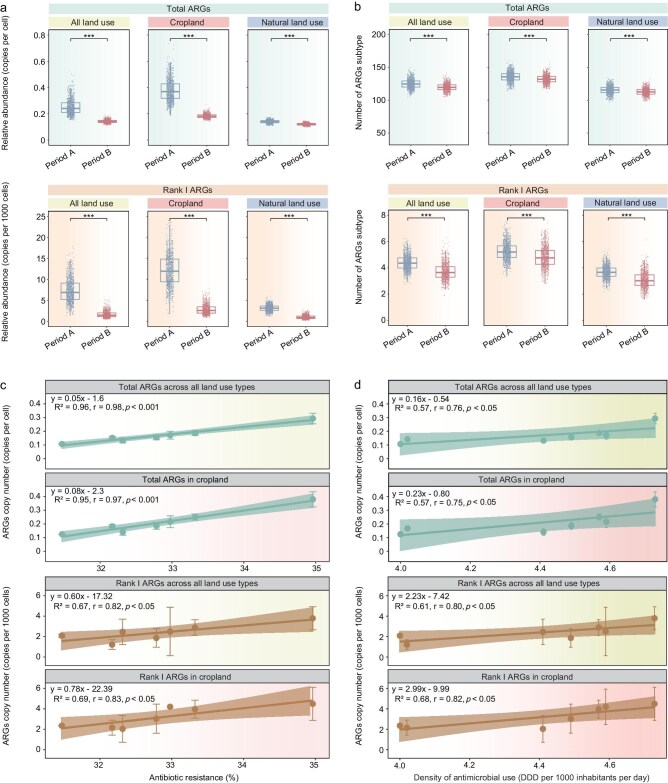
Temporal changes in the relative abundance and risk of soil ARGs in Chinese soils. (a) Temporal changes in the relative abundance of total ARGs and Rank I ARGs in Chinese soils. The samples were divided into two periods based on time, including Period A (before 2015, 2009–2015) and Period B (after 2016, 2016–2022). Temporal trends were calculated across the three land use groups, including total land use type, cropland and natural land use type. Total land use type included all land uses. The grassland, forest and unutilized land were classified into natural land use type. In the comparison of total land use types, 999 rounds of sampling were performed based on the stable sample size in the stable land use types (cropland: 90, forest: 30, grassland: 30, unutilized land: 10, urban: 10) for the calculation of both total ARGs and Rank I ARGs. In the comparison of cropland and natural land use types, 999 rounds of sampling were performed based on the stable sample size (cropland: 70, natural land use type: 70). In the boxplots of panels, hinges indicate the 25th, 50th and 75th percentiles, whiskers indicate 1.5× interquartile ranges and dots indicate values of individual samples. (b) Temporal changes in the diversity of total ARGs and Rank I ARGs in Chinese soils. The samples were divided into two periods based on time, including Period A (before 2015, 2009–2015) and Period B (after 2016, 2016–2022). Temporal trends were calculated across the three land use groups, including total land use type, cropland and natural land use type. Total land use type included all land use types. The grassland, forest and unutilized land were combined into natural land use type. In the boxplots of panels, hinges indicate the 25th, 50th and 75th percentiles, whiskers indicate 1.5× interquartile ranges and dots indicate values of individual samples. (c) The relationship between the relative abundance of ARGs and Rank I ARGs in China and Chinese clinical antibiotic resistance (from 2015–2022). Total 2016 samples were excluded due to the limits of sample size (14 samples). Total 999 rounds of sampling were performed based on the minimum sample size (cropland: 15, forest: 5, grassland: 2, unutilized land: 3, urban: 7) for the calculation of both total ARGs and Rank I ARGs. Antibiotic resistance was calculated as the mean value based on the 13 collected pathogens [15]. (d) The relationship between the relative abundance of ARGs and Rank I ARGs and density of antimicrobial use in China (from 2015–2022). Total 2016 samples were excluded due to the limits of sample size (14 samples). *R*^2^ was calculated from the linear model, and *r* was calculated from the Pearson correlation (linear regression). The correlation was statistically tested using both Pearson’s correlation test (two-sided) and a linear model (two-sided). Gray shading denotes the 95% confidence intervals. Error bars represent standard deviation (SD). Significant comparisons (two-sided *t*-test) between different periods are indicated by ***, *P* < 0.001.

Time series analysis revealed distinct trends between Chinese and global soils. The relative abundance of both total ARGs and Rank I ARGs in Chinese soils significantly decreased over time (*P* < 0.001, *r* = −0.72 to −0.78), whereas, in the global samples, only Rank I ARGs showed a significant increasing trend with time (Figs S3 and S5). To confirm the reliability of the results, further analysis was conducted based on different land use type groups of Chinese soil, including All (samples for all land use types), Cropland (cropland samples) and Natural land use type (soil samples of forest, grassland and unutilized land), with 999 iterations of subsampling. The results revealed a consistent trend across different land use type groups (Fig. 4a and b). Briefly, for Chinese soils, a significant decrease in the relative abundance (Fig. 4a) and diversity (Fig. 4b) of total and Rank I ARGs was observed in Period B compared to Period A (Data S8).

The relative abundance of total ARGs in the All soil group declined from 0.25 to 0.14 copies/cell (∼44.1%) and Rank I ARGs from 7.3 to 1.7 copies/1000 cells (∼76.7%) (Fig. 4a). The decline was more pronounced in Cropland group, where the total ARGs decreased from 0.38 to 0.18 copies/cell (∼52.6%), and Rank I ARGs dropped from 12.2 to 2.8 copies/1000 cells (∼77.0%, Fig. 4a). In Period A, the relative abundance of total and Rank I ARGs in Chinese soils were 1.4–2.8 and 17.1–48.5 times higher than in global soils, respectively. In Period B, these differences narrowed to 1.1–1.6 times (Fig. S6). Meanwhile, diversity followed the overall trend of relative abundance, but its decline was relatively moderate, with reductions of 3.0% for total ARGs and 13.3% for Rank I ARGs in Period B (Fig. 4b). The Cropland group showed the smallest reduction, accounting for 2.7% and 8.3%, respectively. The unrarefied results (Fig. S6) were consistent with those obtained after rarefaction (Fig. 4b). Overall, these results highlighted a significant downward trend in the abundance and risk of soil ARGs in China after implementation of the Plan, with reductions ranging from 44.1% to 77.0%.

To further confirm the influence of Plan on soil resistomes, we examined the relationship between soil resistomes and clinical data, including clinical antibiotic resistance (Fig. 4c, national mean isolation rate of 13 surveyed antimicrobial-resistant pathogenic bacteria during 2015–2022) [15], density of antimicrobial use (Fig. 4d) and veterinary antimicrobials consumption in China [16] (Fig. S7). These indices were directly influenced by the Plan and can serve as indicators of its effectiveness in human sector. Resampling iterations of 999 times were also performed to standardize the number of samples per land use type across years. Indeed, the 2016 sample size was limited (14 samples), but sensitivity analyses showed that including or excluding the 14 samples from 2016 did not substantially change the observed temporal trends in the relative abundance of ARGs or Rank I ARGs (Fig. 4 and Figs S8–S9). Both All soil group and Cropland group were analyzed, and similar results were observed. Results showed that the relative abundance of total ARGs and Rank I ARGs in Chinese soils was significantly correlated with clinical antibiotic resistance, density of antimicrobial use and veterinary antimicrobials consumption (*P* < 0.05, *R*^2^ = 0.58–0.96) (Fig. 4c and d and Fig. S7). According to the linear model results, for every 0.1 DDDs reduction in the density of antimicrobial use, total ARGs decrease by 0.02 copies/cell, while Rank I ARGs decrease by 0.2–0.3 copies/1000 cells. For every 1000-ton reduction in veterinary antimicrobial consumption, total ARGs decrease by 0.01 copies/cell, and Rank I ARGs decrease by 0.07–0.08 copies/1000 cells. Overall, these results suggested that soil ARGs were closely aligned with clinical resistance and antimicrobial consumption (both clinical and veterinary), highlighting the strong linkage between the soil resistome and clinical settings.

### Environmental drivers of ARGs in Chinese croplands

**Figure 5.**
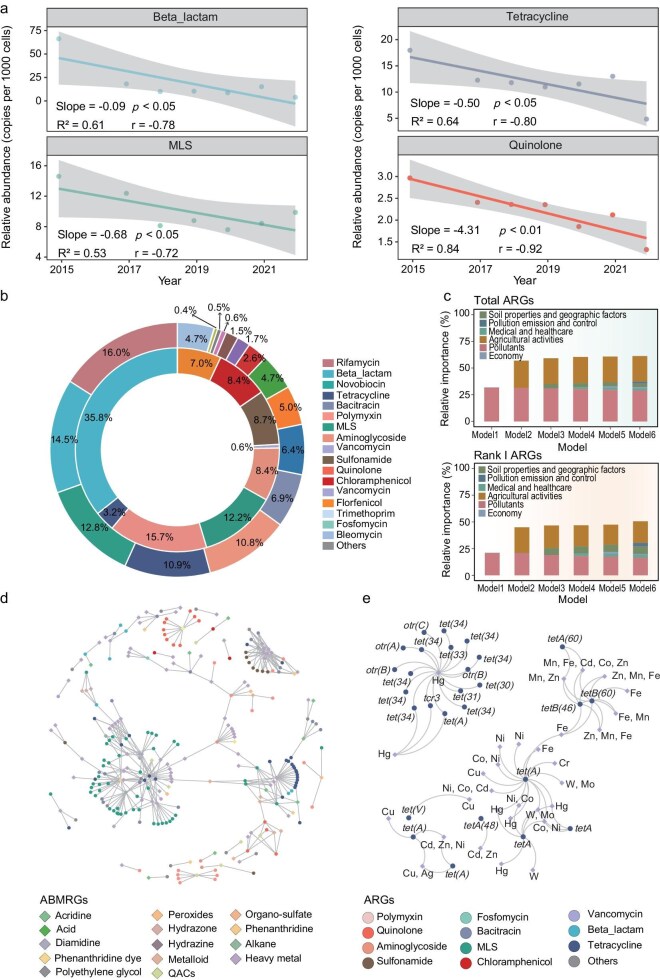
The impact of co-selection of pollutants on ARGs in Chinese cropland. (a) ARG types significantly reduced between 2015 and 2022. The classification criteria and complete results are shown in Figs S8 and S9. Total 2016 samples were excluded due to the limits of sample size (14 samples). Total 999 rounds of sampling were performed based on the minimum sample size (cropland: 5, forest: 5, grassland: 5, unutilized land: 4, urban: 5) for the calculation of both total ARGs and Rank I ARGs. *R*^2^ was calculated from the linear model, and *r* was calculated from the Pearson correlation (linear regression). The correlation was statistically tested using both Pearson’s correlation test (two-sided) and a linear model (two-sided). Gray shading denotes the 95% confidence intervals. (b) The profile of soil ARGs for samples collected in 2021. Different colors represent the various ARGs types. The outer circle of the pie chart represents the proportion of various ARGs types. The inner circle of the pie chart represents the proportion of various Rank I ARGs types. (c) Key factors driving changes in total ARGs and Rank I ARGs in cropland. The used indexes were shown in Data S10. (d) The co-occurrence frequency of ARGs and ABMRGs on the same contigs. QACs, quaternary ammonium compounds. (e) ARGs related to tetracycline and ABMRGs related to heavy metal resistance gene subnetwork. Nodes represented individual ARG or ABMRG variants (only co-occurrences between ARGs and ABMRGs were considered). Edges represented their co-localization on the same contigs.

Linear model analysis was performed to investigate the impact of the Plan on the temporal trends of ARG types (excluding 2016), based on rarefied data from 999 iterations. The trends of 29 ARGs types were classified into six categories—significantly decreased, decreased, fluctuated, stabilized, increased and significantly increased—based on *P* values, slope and the coefficient of variation (Data S9 and Fig. S10). A total of 10 ARGs types exhibited a decreasing trend, among which 6 ARGs types were significantly decreased, including beta-lactam (*P* < 0.05, *R*^2^ = 0.61), MLS (*P* < 0.05, *R*^2^ = 0.53), quinolone (*P* < 0.01, *R*^2^ = 0.84) and tetracycline (*P* < 0.05, *R*^2^ = 0.64) (Fig. 5a). These ARG types correspond to the antibiotics with the highest consumption intensity in clinical (beta-lactam and quinolone), and veterinary (tetracycline and MLS). No ARG types showed a significantly increasing trend (Fig. S11), likely attributed to the implementation of the Plan.

Spanning the transition from the Plan (2016–2020) to the next phase (2022–2025), 2021 serves as a key point to assess the previous plan’s impact and set a baseline for the following phase. Given the importance of cropland, 110 soil samples were collected across China in July 2021. The abundance of ARGs in these samples reached 0.14 copies/cell, with high-risk ARGs reaching 2.0 copies/1000 cells. We found beta-lactam (35.8%), polymyxin (15.7%) and MLS (12.2%) dominated among Rank I ARGs (Fig. 5b). Given their continued presence in croplands, these ARGs types should be prioritized for further monitoring, control and management.

To uncover the driving forces behind ARGs persistence, we collected diverse datasets to investigate key factors influencing the soil resistome in cropland, including soil physicochemical properties, economic indicators, agricultural activities, pollutant emissions and clinical and healthcare data. As complex pollution in soil was another significant issue, a comprehensive contaminant screening detected 19 of 31 antibiotics, all 12 tested metals, 34 of 375 pesticides and 9 of 19 non-antibiotic pharmaceuticals (Fig. S12). After removing collinear variables (used indices were shown in Data S10), ridge regression model was conducted and showed that pollutant concentrations significantly influenced the residual total ARGs (29.0%–31.7%) and Rank I ARGs (16.4%–20.9%) in cropland soil (Fig. 5c). Meanwhile, agricultural activities also made substantial contributions to the variation in total ARGs (23.9%–25.4%) and Rank I ARGs (20.3%–24.2%) (Fig. 5c).

Besides antibiotics, ARGs in cropland soils could also be influenced by non-antibiotic compounds. Therefore, we further analyzed the co-occurrence frequency of ARGs and antibacterial biocide and metal resistance genes (ABMRGs) on the same contigs to explore the potential role of co-selection in shaping soil ARGs and constructed a co-occurrence network (Fig. 5d and Data S11). Briefly, a total of 1598 co-localization events between ARGs and ABMRGs were observed. These events involved 160 ARGs variants (including 26 Rank I ARGs), 115 ABMRGs variants and 374 network edges. The ARGs variants spanned 19 types, including tetracycline (532 events), MLS (317 events), bacitracin (180 events), novobiocin (135 events), sulfonamide (122 events) and quinolone (115 events). The ABMRG variants conferred resistance to heavy metals (1076 events), aromatic hydrocarbons (189 events), metalloids (50 events) and quaternary ammonium compounds (45 events). The subnetwork of metal-related ABMRGs and tetracycline-related ARGs highlighted that tetracycline ARGs frequently co-occurred with resistance genes for metals in cropland soils (Fig. 5e). This co-occurrence was particularly pronounced for genes associated with Mn, Fe, Cd, Co, Zn and Cu resistance, suggesting that elevated concentrations of these metals may exert potential selective pressure on tetracycline ARGs. Overall, the results suggest that chemical pollutants, particularly non-antibiotic compounds, may play an important role in the co-selection of ARGs, serving as a key factor shaping the soil resistome and undermining the effectiveness of the Plan.

### Connectivity between shared ARGs in Chinese and global soil samples

In addition to chemical pollutants, how agricultural activities shape the composition and connectivity of soil ARGs remains unclear, despite their substantial contribution to antibiotic resistomes. First, the profile-based analysis showed that all core ARGs subtypes (nine subtypes, accounting for 52.0%) identified in global soils (Data S12, excluding Chinese samples) were also classified as core ARGs in Chinese soils (Fig. S13). The relative abundance of these core ARGs in cropland was 1.3–1.5 times higher than that in natural land use types (Data S3 and Data S12). This similarity might be attributed to the combined effects of antibiotic and ARG inputs from agricultural activities. Meanwhile, β-diversity of ARGs profile was significantly lower in cropland than in natural soil (*P* < 0.001, Fig. S14), demonstrating that the antibiotic resistomes in cropland soils are more similar than those in paired natural land use types.

**Figure 6.**
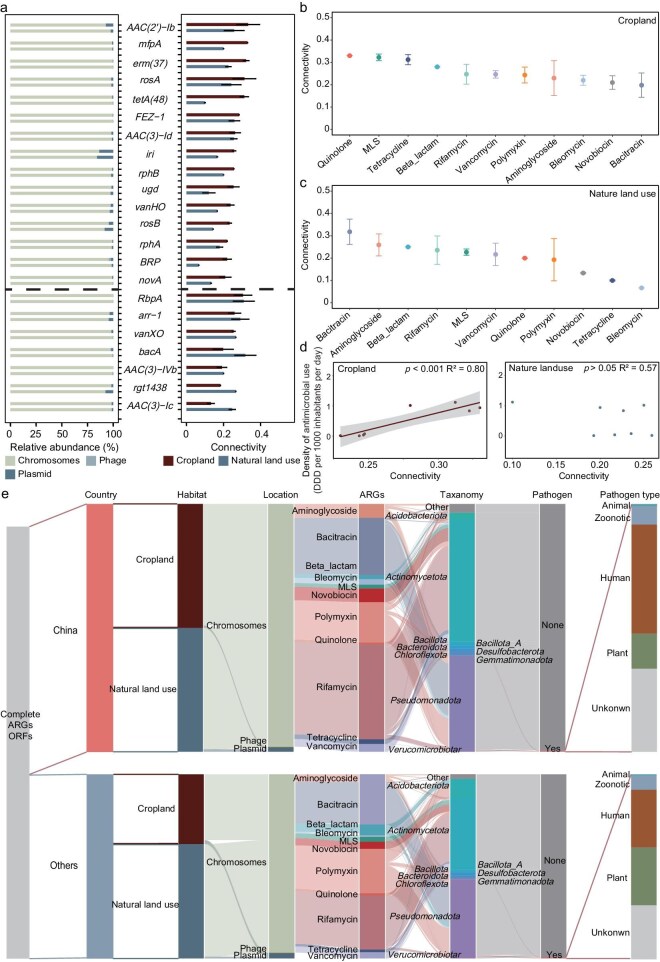
ARGs connectivity between China and global samples. (a) The ARGs subtypes included in connectivity calculation and their locations. The grassland, forest and unutilized land were combined into natural land use type. Only complete ARG ORFs were included in the analysis. For each identical ARG subtype, 50 sequences from cropland and natural group in both Chinese and global soils were randomly subsampled 999 times. Subtypes positioned above the dashed line indicate higher connectivity in croplands, while those below the line indicate higher connectivity in natural land uses. BRP: bleomycin resistance protein. (b) The connectivity of ARGs type in cropland. The connectivity of each ARGs type was calculated as the mean value of the subtypes included within that type. Subtypes positioned above the dashed line indicate higher connectivity in croplands, while those below the line indicate higher connectivity in natural land uses. (c) The connectivity of ARGs type in natural land use type. The connectivity of each ARGs type was calculated as the mean value of the subtypes included within that type. Error bars represent SD. (d) The relationship between the connectivity of ARGs type and density of antimicrobial use in cropland (left) and natural land use types (right). The density of antimicrobial use for each antimicrobial was calculated as the mean value for each antimicrobial type based on the global antimicrobial use data (2016 to 2022, https://worldhealthorg.shinyapps.io/glass-dashboard/#!/amu). Pearson correlation test (two-sided) was conducted. Gray shading denotes the 95% confidence intervals. (e) Profile of ARGs carrying contigs in Chinese and global soils, including habitats, ARGs carriers, ARGs location, taxonomy (phylum), pathogenicity and risk level. Only ARGs located on chromosomes were used for subsequent taxonomy analysis. Only ORFs included in the connectivity analysis were included.

We next performed a phylogeny-based analysis to compare the global connectivity of ARGs subtypes between cropland and natural soils (Fig. 6 and Text S2). All metagenomic datasets were assembled, and complete open reading frames (ORFs) annotated as ARGs were extracted for further analysis. A total of 22 ARGs subtypes with >50 ORFs detected in both cropland and natural soils from Chinese and global samples were included in the analysis (Fig. 6a). These selected ARGs encompassed the majority of core ARGs in both Chinese (13/15, 80%) and global soils (9/9, 100%) (Fig. S13, and Data S3 and Data S12), accounting for 53.0% to 63.2% of the relative abundance of total ARGs. Meanwhile, these ARGs made up 61.9% of the total in Chinese cropland soil samples collected in 2021 (Data S13). Thus, these ARGs could represent the core antibiotic resistome. Their average connectivity was higher in cropland (connectivity = 0.27) than in natural land use type (connectivity = 0.20). Among them, 15 subtypes showed higher connectivity in cropland soils, and no correlation was observed between mobility and connectivity (Fig. 6a). These results suggested that, among these 22 ARGs subtypes included in the analysis, ARGs connectivity tends to be higher in cropland soils, which may indicate a trend toward homogenization.

Moreover, the ARGs types with high connectivity differed substantially between cropland and natural land use type (Fig. 6b). Specifically, beta-lactam, quinolone, MLS and tetracycline ARGs exhibited higher connectivity in cropland soils, whereas bacitracin, aminoglycoside and rifamycin ARGs showed higher connectivity in natural land use type (Fig. 6b). We found that the connectivity of ARGs significantly associated with global density of antimicrobial use (Fig. 6e) but not with the year of antibiotic introduction (Fig. S15), showing a positive correlation in cropland (*P* < 0.001, *R*^2^ = 0.80).

Finally, to identify the hosts of these ARGs, we only focused on those located on chromosomes (97.5%), given the challenges in resolving plasmid- (2.3%) and phage-associated ARGs (0.2%) (Fig. 6a). A total of 54 pathogenic species carrying these ARGs were identified, with the most common being *Escherichia coli* (human), *Pectobacterium carotovorum* (plant), *Acinetobacter johnsonii* (human), *Pantoea agglomerans* (plant) and *Achromobacter xylosoxidans* (zoonotic). Together, the homogenization and high connectivity of core ARGs in croplands highlight the influence of agricultural activities on soil resistomes and raise concerns that unregulated practices could undermine ongoing AMR mitigation efforts.

### Open-access interactive map for Chinese soil ARGs

To better understand and monitor the spatiotemporal distribution of ARGs in Chinese soils, we developed an open-access interactive map based on the 2243 Chinese soil metagenomic datasets (from 2009 to 2022). Specifically, the website supports individual retrieval and quick display of profiles for samples based on land use type, time and province. The abundance and diversity of ARGs and Rank I ARGs for each year and region were also compiled. Additionally, the platform allows for the retrieval of specific ARGs types, subtypes and variants, providing an efficient way to track the appearance of different ARGs in Chinese soils over time and across locations. As some efflux pumps are functionally linked to AMR, multidrug resistance genes were retained in the website dataset to provide a more comprehensive resource for users, allowing flexible selection depending on needs. The website is temporarily accessible at https://smile.hku.hk/map/.

## DISCUSSION

Based on 2243 soil metagenomic datasets from China, we constructed a nationwide interactive map of soil ARGs in China and developed an open-access platform to visualize and explore data collected between 2009 and 2022. Our results showed a national-scale decline in the relative abundance of total ARGs (52.6%) and Rank I ARGs (77.0%; *P* < 0.001) between the periods before 2015 and after 2016, which was temporally associated with the implementation of AMR control policies. However, the persistent presence and high diversity of ARGs in croplands remain major challenges, largely driven by chemical co-selection and the convergence of core ARGs under agricultural activities.

According to the profile-based analysis, we found that cropland exhibiting the highest relative abundance (1.7–1.8 times), the highest risk (3.1–6.9 times) and the greatest diversity (1.2–2.5 times) compared to other land use types (Fig. 2). These results were also observed in the global dataset (Fig. 4a) and were consistent with another study using 1088 samples (global) [9], which found the highest ARGs abundance in cropland soils. A total of 107 Rank I ARGs subtypes were identified in cropland soils (Fig. 3), and the widespread presence of plasmid-mediated ARGs, including *mcr, bla*_NDM_ and *CTX-M*, was observed [20]. These ARGs exhibited both high clinical relevance and presence in cropland soils, suggesting that they may have crossed ecological boundaries and potentially disseminated from human sources to cropland soils [21]. Beyond direct introduction via generalist taxa [22], the persistence of Rank I ARGs may be promoted by vertical gene transfer and stress-induced horizontal gene transfer (HGT), along with antibiotic residues and co-selection from diverse pollutants [23]. Plasmids and phages may act as key mediators of HGT, facilitating the dissemination of ARGs across microbial communities, particularly in soils contaminated with pesticides [24] or microplastics [11]. Moreover, significant differences could be observed between the Rank I ARGs subtypes present in China and globally. Chinese cropland soils harbored a greater number and higher relative abundance of unique and significantly enriched Rank I ARGs subtypes (4.6 copies/1000 cells), which was 7.7 times higher than global cropland (0.6 copies/1000 cells) (Fig. 4). These results indicated that the environmental dissemination of Rank I ARGs still exhibited regional disparities.

Among the surveyed Chinese soil samples, 6 *mcr* subtypes and 1 *NDM* subtype were identified, including, *bla*_NDM-19_ [25] (2017, *Klebsiella pneumoniae*), *mcr-1.7* [26] (2016, *E. coli*), *mcr-3.19* [27] (2016, *E. coli*) and *mcr-3.20* [28] (2016, *E. coli*). Most of these ARGs are clinically important ARGs against polymyxins and carbapenems that were first reported recently from clinical or animal-derived samples in China. The absence of detection does not equate to true absence. This may be due to limited sample size, uneven geographic coverage or misalignment between reported detection sites and actual ARGs origins. *bla*_NDM-1_ is a typical example. Although it was first reported in a Swedish patient, the patient had received medical treatment in India [29]. Moreover, the time of the first discovery of ARGs may differ from its actual period of circulation. For example, although *mcr-1* was initially reported in 2015 [30], retrospective analyses revealed that >15% of *E. coli* isolates carried *mcr-1* between 2011 and 2014 [31]. Given that all currently available reference sequences originate from human isolates, the presence of *bla*_NDM-19_ and *mcr-1.7* in post-2016 soil samples may suggest spillover from the human/clinical source (sequence and isolated source were shown in Data S14). Due to the lack of veterinary or environmental reference sequences, we cannot rule out additional sources, but the available data indicate a potential clinical origin.

Indeed, previous studies have elucidated various aspects of the global soil resistome [8–10]. Yet, the lack of region-specific investigations may conceal spatial heterogeneity and underestimate the true risk posed by soil ARGs in local ecosystems. Our study offers the most comprehensive profile of soil ARGs in China to date, based on 2243 metagenomic datasets (including 859 from cropland). As extensive regional datasets better reveal the presence of clinically relevant ARGs in soil, we recommend strengthening surveillance and sampling in priority regions.

Our results showed a temporal decline in soil ARGs abundance and potential risk in China, particularly in croplands, where total ARGs and Rank I ARGs decreased by 52.6% and 77.0%, respectively, from Period A to Period B (Fig. 4a and b). Over 70% of ARGs subtypes exhibited a decrease, with quinolones, beta-lactams, tetracyclines and MLS showing the most pronounced reductions (Fig. 5a). These downward trends coincided with national declines in clinical antibiotic resistance, antimicrobial use and veterinary antimicrobial consumption (Fig. 4c and d and Fig. S6).

Considering the association between soil, human and livestock resistomes [8], the observed reductions might be related to the implementation of stricter antimicrobial regulations under the Plan (2016–2020). Key measures of the Plan included stricter regulation of clinical and veterinary antibiotic use and restriction of human–veterinary shared antibiotics. Meanwhile, environmental interventions, such as improved management of pharmaceutical waste and monitoring of soil and water contamination, could reduce the introduction of ARGs into cropland soils. These measures could plausibly influence microbial community and ARGs abundance, providing a potential explanation for the observed decrease. It should be noted that our results reflect temporal associations rather than definitive causal relationships. In the absence of controlled experimental or counterfactual data, the observed temporal trends may also have been influenced by other concurrent factors, including the implementation of environmental policies (e.g. Water Pollution Prevention and Control Action Plan and Air Pollution Prevention and Control Action Plan), changes in cropping practices and decreases in other anthropogenic inputs.

Despite these achievements, the detection of previously unreported ARGs (Fig. 1), residual ARGs (Fig. 5) and the limited decline in ARGs diversity (3.0%–13.3%, Fig. 4), especially in croplands, remain pressing challenges. Undoubtedly, advances in metagenomic sequencing technologies and increased sequencing depth have contributed to this observation [32]. Rarefaction analyses of soil metagenomes reached saturation, indicating that the selected samples sufficiently captured the Chinese soil resistome (Fig. 2a). Various analyses consistently indicated that pollution legacies and agricultural activities constitute major barriers to further reductions in soil ARGs (Figs 5 and 6). Chemical pollutants (e.g. metals) could exert co-selection pressure on ARGs (tetracycline resistance genes) by selecting for the corresponding metal(loid)s/pesticide resistance genes or genes related to efflux pumps, and through co-selection of species and genes, contribute to the increased abundance and spread of ARGs [33]. Widely used antibiotics such as beta-lactam and tetracycline, along with their corresponding ARGs, may enter cropland soils through various pathways [34].

Moreover, based on the ARGs subtypes included in the connectivity analysis, we found that agricultural activities may drive the global homogenization of soil ARGs at both the profile (β-diversity) and phylogenetic levels (Fig. S12 and Fig. 6). Although the surveyed ARGs subtypes are representative of the core soil resistome, accounting for >50% of the total ARGs relative abundance and covering 80% of core ARGs subtypes, the total number of the surveyed subtypes remained limited (*n* = 22). It is possible that some of these core ARGs might reflect intrinsic soil resistance [9]. The robustness of connectivity analyses could be further improved by incorporating a broader range of ARG subtypes in future studies. This phenomenon observed in soil resistomes was consistent with ecological theories of microbial community convergence in croplands [23]. Agricultural activities may promote the formation of a globally convergent core soil resistome through continuous direct inputs (e.g. antibiotics and ARGs) [34,35], indirect selection through shaping of microbial communities [36,37] and HGT [38]. Notably, this global convergence suggested that intensive agricultural practices can erode natural biogeographic patterns and generate highly connected resistome networks that transcend national boundaries [9,39]. The observed high connectivity may be partly explained by convergent selection under similar agricultural pressures globally, including manure application, reuse of treated wastewater, antibiotic residues and fertilizer application, while potential contributions from direct cross-border transmission cannot be excluded. Plausible physical pathways include hydrological transport, airborne dispersal, trade of livestock and crops, human movement and engineered interventions, underscoring the need for coordinated international action [40,41]. Due to the limitations of short-read sequencing, quantitatively assessing how specific agricultural practices influence ARG connectivity in individual samples remains a key scientific challenge.

Remarkably, a substantial number of identical ARGs variants have been detected in *E. coli* isolated from soil and human [42], suggesting the high connectivity between soil and human resistome [8]. Direct inhalation, consumption of contaminated food crops and groundwater exposure might represent potential pathways linking soil to human resistomes [43]. Therefore, future mitigation strategies should move beyond local interventions and adopt globally coordinated actions to standardize agricultural management practices and curb the convergence and spread of ARGs. Key measures include integrated control of chemical pollutants in croplands, reduced antibiotic inputs, prohibition of untreated manure and wastewater application and promotion of standardized and regulated agricultural practices [44]. These findings highlight the necessity of integrating environmental connectivity metrics into AMR risk assessment frameworks and of implementing coordinated international strategies to manage agricultural ARG dissemination. We acknowledge the limitation that the lack of data from LMICs may have led to an underestimation of soil ARGs levels in some global regions, highlighting the need for more comprehensive sampling and surveillance in LMICs. In addition, although sequence similarity-based approaches are widely applied, this approach may introduce uncertainty, including potential false positives and false negatives, particularly for genes with limited representation in databases. Finally, the sample size collected in this study may not fully represent the distribution of ARGs in soils across China, given the high diversity of soil types and the substantial variation in environmental conditions across regions. Additional sampling, particularly through systematic coverage of different soil types and ecological regions, could enhance the representativeness and generalizability of the results.

## METHODS

### Collection of public soil metagenomic data

To minimize potential bias, only public metagenomic data meeting the following criteria were included for analysis (Text S1). Briefly, a total of 2133 Chinese public soil samples were collected (Data S1). To assess differences between China and other countries and to calculate the global connectivity of ARGs, we incorporated 2234 soil metagenomic samples from other countries, reported in previous studies [8], into our analysis. To ensure balanced representation of global samples between Period A and Period B, an additional 322 samples were included (Data S6). Overall, 4689 public soil samples were collected, including 2133 Chinese soil samples and 2556 soil samples from other countries. The country, land use type and soil type information, as determined using ArcGIS (v10.8), can be found in Data S1 and S6.

### National metagenomic data sampling

Considering that 2021 falls between the first (2016–2020) and second (2022–2025) phases of the Plan, 2021 provides an opportunity to assess the outcomes of the previous plan while offering baseline data for the next phase. Therefore, we collected 110 cropland soil samples from across China during this year, as cropland was predicted to be the land use type with the highest ARGs abundance [9,45]. The collection methods were previously described [8,46]. We performed a unified analysis on all datasets to eliminate discrepancies arising from different analytical approaches following the methods in our previous studies [8] (Text S1). The data were divided into two periods: Period A (2009–2015), before the implementation of the Plan (2016), and Period B (2016–2023), after the Plan’s initiation. We employed a phylogeny-based approach to identify highly connected ARGs subtypes (Text S2). The connectivity of each ARGs type was calculated as the average connectivity of its subtypes. The antimicrobial consumption intensity was calculated as the mean value of each antimicrobial type from Global Antimicrobial Use data (2016–2022). The construction of Ridge regression model could be observed in Text S3.

### Datasets for Chinese clinical antibiotic resistance

The Chinese clinical antibiotic resistance and antimicrobial consumption intensity (DDDs per 100 inhabitants per day) was collected from https://www.carss.cn/sys/Htmls/dist/index.html. The annual average data (from 2015 to 2022) used were the mean values of these 13 clinically prioritized resistant pathogens’ resistance profiles across different provinces in China for each year.

### Detection for chemical pollutants

We analyzed chemical pollutants in 110 cropland soil samples collected across China in 2021, including 31 antibiotics, 12 heavy metals, 375 pesticides and their residues and 19 non-antibiotic pharmaceuticals. The detected detail could be observed in Text S4.

## Supplementary Material

nwag387_Supplemental_Files

## Data Availability

The in-house metagenomic sequencing data generated in this study have been deposited in the National Center for Biotechnology Information (NCBI) Sequence Read Archive (SRA) database under accession number PRJNA1229199 (https://www.ncbi.nlm.nih.gov/bioproject/PRJNA1229199). All online metagenomic sequencing data used in this study are available in the NCBI RefSeq database, IMG/M portal and European Nucleotide Archive. Information for all metadata used in this study as well as the important data for analysis are provided in Data S1 and S6.
