# Supplementary material for: Environmental effectiveness of the National Action Plan to Contain Antimicrobial Resistance: evidence from Chinese soil
Source: Natl Sci Rev. 2026 Jun 24;13(14):nwag387. doi: 10.1093/nsr/nwag387 (PMC13386501; doi:10.1093/nsr/nwag387)
Supplement: nwag387_Supplemental_Files [file nwag387_supplemental_files.zip › Supplymentary material.docx]

**Environmental Effectiveness of the National Action Plan to Contain Antimicrobial Resistance: Evidence from Chinese Soil**

Yuxiang Zhao^a, b, c#^, Zishu Liu^b, c, d#^, Xi Chen^a^, Yue Huang^a^, Shuxian Li^a^, Xuemei Mao^a^, Xiawan Zheng^a^, Xiangwu Yao^b^, Baolan Hu^b, c, e*^ , Lizhong Zhu^b, c*^, Tong Zhang^a, f, g, h, i*^

^a^ Environmental Microbiome Engineering and Biotechnology Laboratory, Center for Environmental Engineering Research, Department of Civil Engineering, The University of Hong Kong, Hong Kong SAR, China

^b^ State Key Laboratory of Soil Pollution Control and Safety, Zhejiang University

^c^ College of Environmental and Resource Sciences, Zhejiang University, Hangzhou, China

^d^ College of Natural Resources and Environment, Northwest A&F University, Shaanxi 712100

^e^ Zhejiang Key Laboratory of Water Pollution Control and Water Ecological Health, Zhejiang University, Hangzhou, China

^f^ School of Public Health, The University of Hong Kong, Hong Kong SAR, China

^g^ Department of Environmental Science and Engineering, Macau University of Science and Technology, Macao SAR, China.

^h^ The University of Hong Kong Shenzhen Institute of Research and Innovation, Shenzhen, China

^i^ The State Key Laboratory of Marine Environmental Health (SKLMEH), City University of Hong Kong, Hong Kong SAR, China

^*^For correspondence

[Tong Zhang, E-mail:](mailto:zlz@zju.edu.cn) [zhangt@hku.h](mailto:blhu@zju.edu.cn)[k](mailto:zlz@zju.edu.cn)

[Lizhong Zhu, E-mail: zlz@zju.edu.cn](mailto:zlz@zju.edu.cn)

[Baolan Hu, E-mail: blhu@zju.edu.cn](mailto:zlz@zju.edu.cn)

[# These authors contributed equally.](mailto:zlz@zju.edu.cn)

**Text S1 Metagenomic data analysis**

The following criteria were included for analysis: (i) Only soil samples from croplands or natural landuse were included. Samples described in the original studies as originating from obviously polluted environments, including mining sites, coke plants, industrially contaminated soils, or landfills were excluded, (ii) Illumina shotgun paired-end data in FASTQ format, (iii) metagenomic sequencing data, (iv) no culturing or additional experiments were conducted before DNA extraction, (v) read lengths longer than 100 base pairs, (vi) included accurate sampling coordinates, (vii) balanced sample sizes between China and global groups (excluding Chinese samples). These data were collected via Aspera (https://www.ibm.com/cn-zh/products/aspera).

Briefly, low quality reads were removed firstly, and the clean data were obtained using Trimmomatic (v.0.36) [1]. ARGs-OAP (v3.2.2) was used to obtain the ARGs profiles, applying a threshold of 80% identity, 75% coverage, and an e-value of 1e-7 [2]. The SARG-S database, the default database for ARGs-OAP (v3.2.2), was used for comprehensive analysis of environmental metagenomic data through similarity search algorithms, excluding sequences related to point mutations, transcriptional regulators, and other factors [2]. Additionally, to avoid potential mis-annotations of ARGs, genes associated with multidrug efflux pumps were excluded. The effect of fungi on the ARGs-OAP analyses was excluded. The identified ARGs were classified at three levels, including type (also named class), subtype (gene), and variant levels (reference sequence). We used copies of ARGs per cell as the unit for normalization, a consensus unit that accounts for ARGs lengths, sequencing depth, and the number of prokaryotic cells in the dataset [3]. In detail, ARGs copy numbers were estimated by mapping sequencing reads to SARG database and normalizing by gene length. The number of prokaryotic cells were estimated using universal single-copy marker genes. Finally, the normalized ARGs copy numbers was divided by the estimated prokaryotic cell numbers. The copy / cell value was then calculated by dividing the normalized ARGs copy numbers by the estimated cell number. The copy / cell is a quantification unit for environmental ARGs surveillance [3].

$$\frac{\text{copies}}{\text{cell}}\text{=}\frac{\text{1}}{\text{cell}\text{ }\text{number}}\text{∗}\sum\frac{\text{R}\text{eads}\text{ }\text{counts}\text{∗}\text{mapped}\text{ }\text{lengt}\text{h}}{\text{gene}\text{ }\text{lengt}\text{h}}$$

We used the relative abundance of Rank I ARGs to reveal the potential ARGs risks. The Rank I ARGs were proposed based on host pathogenicity, gene mobility and human-associated enrichment, which are the focus of One Health [4]. The profile of Rank I ARGs was determined based on a previously established list (Supplementary Data 2). Briefly, ARGs profiles were first generated for all metagenomic samples, and these were then compared against the predefined Rank I ARGs list to identify the presence of specific variants. The resulting Rank I ARG profiles therefore reflect their occurrence across the sampled metagenomes in this study, while their classification follows prior reports (Supplementary Data 2).

All the metagenomic datasets were assembled by MEGAHIT (v1.2.9) [5]. The ORFs of contigs were predicted using Prodigal (v2.6.3) [6] and only complete ORFs were analyzed. To identify ARGs-carrying contigs, the complete ORFs were aligned to SARG-S using Diamond blastp (v2.1.8.162) [7]. After extracting the contigs carrying complete ARGs ORFs, geNomad was used to differentiate plasmids and viruses [8]. The remaining contigs were annotated using Kraken2 with GTDB (release 220) [9], excluding those identified as plasmids and viruses. ARGs-carrying prokaryotic pathogens were identified as previously described [10] (listed in Supplementary Data 17). To identify ABMRGs-carrying contigs, the complete ORFs were aligned to antibacterial biocide and metal resistance genes database using Diamond blastp (v2.1.8.162) [11]. All complete ORFs were compared against antibacterial biocide and metal resistance genes database [11] (BacMet) using Diamond blastp (v2.1.8.162), and sequences co-occurrence with ARGs on the same contigs were considered as co-occurring ARGs and metal resistance genes.

**Text S2 The connectivity of ARGs between China and global samples**

All complete ORFs were extracted, grouped by subtypes, and labeled by country group (China and global). To include more ARGs in the analysis, we divided land use types into cropland and natural land (i.e., forest, grassland, and unutilized land) use types. ARGs subtypes included in the analysis were required to have more than 50 complete ORFs in both the cropland and natural land use types in China and globally. For each identical ARG subtype, 50 sequences from cropland and natural land use types in both China and global soils were randomly subsampled 999 times. Complete ARGs ORFs were aligned using ClustalW (v2.1), and phylogenetic trees were constructed using FastTree (v2.1). Connectivity was assessed by measuring the frequency of group (China and global) transitions between neighboring positions, and the values were normalized relative to the theoretical minimum (complete differentiation, actual connectivity = 2) and maximum (fully connected, actual connectivity = 100).

**Text S3 Ridge regression model**

Ridge regression, an L2-regularized form of multiple linear regression was applied to quantify the contributions of different explanatory variable groups to the variation in Rank I ARGs. All analyses were conducted in Python (version 3.10.12) using the pandas library (version 2.2.2) and the scikit-learn library (version 1.5.1). Six groups of predictors were predefined based on their environmental and socio-economic relevance: pollutants, agricultural activitie, soil physicochemical properties, clinical and healthcare data, pollutant emissions, and economic indicators. A total of 375 factors were collected from China Statistical Yearbook 2022. Prior to model fitting, we assessed multicollinearity among predictors by calculating the variance inflation factor (VIF) for each variable. Variables with VIF values exceeding the threshold of 10 were iteratively removed until all remaining predictors met the criterion. After removing multicollinearity factors, a total of 18 factors were included in further analysis, including pollutants concentration (non-antibiotic drugs, metal, antibiotic, pesticides and pesticide residues), agricultural activities (total manure land application rate, total livestock manure production, compound fertilizer, mulching film coverage area, and number of household biogas figesters), soil properties and geographic factors (longitude and elvation), clinical and healthcare data (antibiotic use rate in tertiary hospitals, antibiotic use rate in secondary hospitals, antibiotic consumption intensity), pollutant emissions (domestic chemical oxygen demand emissions and domestic ammonia nitrogen emissions), and economic indicators (gross regional product and natural growth rate). The ridge regression model was fitted sequentially by incrementally adding groups of predictors in the predefined order. For each step, a ridge regression model was trained with a regularization parameter α = 1.0 using the Ridge() function from sklearn.linear_model. Model performance was evaluated by calculating the coefficient of determination (R²) using the r2_score() function. To assess the relative contribution of each group within a model, feature coefficients were extracted, and the absolute values of coefficients were summed within each group. Group contributions were expressed as percentages of the total absolute coefficient sum in the model. The explained variance (R²) attributable to each group was then calculated by multiplying its percentage contribution by the model R².

**Text S4 Detection for chemical pollutants**

The 12 metals included Al, Fe, Ca, Pb, Cd, Cr, Zn, Cu, Mn, Ni, Hg, and As. The samples were subjected to strong acid digestion using a mixture of concentrated nitric acid (HNO_3_), hydrofluoric acid (HF), and hydrogen peroxide (H_2_O_2_), following a HNO_3_–HF–H_2_O_2_ digestion protocol for total metal analysis in soils. The concentrations of these metals were subsequently determined using inductively coupled plasma mass spectrometry (ICP-MS).

A total of 31 antibiotics covering 9 major classes were analyzed, including carbapenems, penicillins, cephalosporins, sulfonamides, fluoroquinolones, tetracyclines, macrolides, glycopeptides, and quinoxalines. The concentrations of antibiotics were detected as previously described [12, 13]. Soil samples were first freeze-dried and sieved through an 80-mesh screen. 0.5 g of prepared soil was mixed with 25 mL of a solution containing equal volumes of methanol and EDTA-McIlvaine buffer (12.9 g of citric acid monohydrate, 27.5 g of disodium hydrogen phosphate, and 37.2 g of Na_2_EDTA in 1 L of deionized water, with the pH adjusted to 4.0). The mixture was sonicated for 10 minutes, followed by centrifugation at 4000 rpm for 10 minutes. This process was repeated, and all supernatants were combined and diluted with deionized water to a final volume of 500 mL. Solid-phase extraction (SPE) was then performed using Oasis HLB cartridges. The cartridges were conditioned sequentially with 10 mL of methanol, 10 mL of deionized water, and 5 mL of acidified water (pH 5.0 ± 0.2). After sample loading, the columns were rinsed with 10 mL of water and air-dried for 15 minutes. Target compounds were eluted using 10 mL of a 1:1 mixture of methanol and acetonitrile. The eluate was evaporated to dryness under a nitrogen stream, then reconstituted in 0.5 mL of a methanol-water solution (1:1, v/v). After centrifugation at 10,000 rpm for 10 minutes, the clarified supernatant was transferred to vials for analysis. Quantification was carried out using a SCIEX Triple Quad™ 5500 LC-MS/MS system at the Zhejiang Ecological and Environmental Monitoring Center (Hangzhou, China). The limits of detection (LODs) were approximately 0.05–10 ng/g, and the limits of quantification (LOQs) were in the range of 0.5–40 ng/g, depending on the compound and matrix. The recovery rates for each antibiotic ranged from 73.1 - 101.1% [12, 13].

A total of 375 pesticides and pesticide residues were detected. A detail list is available in Supplementary Data 18. The analytical method was established with reference to the Chinese national standard GB 23200.121-2021 and the pesticide standards were purchased from Shanghai Anpel Laboratory Technologies Inc. (Shanghai, China). In detail, soil samples were extracted and cleaned up using a QuEChERS-based method involving acetonitrile extraction, salt-induced partitioning with MgSO_4_ and sodium acetate, followed by cleanup with MgSO_4_, PSA, and C18 sorbents. Quantification of pharmaceutical compounds was performed using an ultra-performance liquid chromatography system (Waters Acquity UPLC) coupled with a triple quadrupole mass spectrometer (AB SCIEX 5500 QQQ-MS) equipped with an electrospray ionization (ESI) source. Chromatographic separation was achieved on an Acquity UPLC BEH C18 column (1.7 µm, 2.1 × 100 mm) maintained at 40 °C. The mobile phase consisted of solvent A (water containing 0.01% formic acid and 2 mM ammonium formate) and solvent B (methanol containing 0.01% formic acid and 2 mM ammonium formate). The flow rate was set at 0.30 mL/min, with an injection volume of 6 µL. The total run time was 30 minutes. The mass spectrometer was operated in ESI mode with the following parameters: curtain gas at 35 psi, collision gas at 7 psi, ion spray voltage of 4500 V, source temperature of 450°C, and ion source gases 1 and 2 both at 35 psi. Data acquisition and quantification were performed using multiple reaction monitoring (MRM) mode. The LOD were approximately 0.5–2 ng/g, and the LOQ were in the range of 1–5 ng/g, depending on the compound and matrix.

A total of 19 non-antibiotic pharmaceuticals were detected, including anti-inflammatory drugs (e.g., ibuprofen, aspirin), antifungals (e.g., ketoconazole, fluconazole), antivirals (e.g., ribavirin, oseltamivir), hormones (e.g., estradiol, testosterone), corticosteroids (e.g., dexamethasone, methylprednisolone), and antidepressants (e.g., sertraline, duloxetine). The analytical standards were purchased from Shanghai YuanYe Bio-Technology Co., Ltd. (Shanghai, China). One gram of the homogenized sample was weighed into a 10 mL centrifuge tube, followed by the addition of 3 mL acetonitrile containing 1% acetic acid and a ceramic homogenization bead. The mixture was vigorously shaken for 1 minute and vortexed for an additional 1 minute. Ultrasonic extraction was then performed at 4 °C for 30 minutes. Afterward, the sample was centrifuged at 4200 rpm for 5 minutes, and the supernatant was collected and evaporated to dryness using a rotary evaporator. The residue was reconstituted in 0.10 mL of a 1:1 (v/v) acetonitrile–water mixture, vortexed thoroughly, and centrifuged again. The resulting supernatant was transferred to an autosampler vial for analysis. Chromatographic separation was achieved using an Acquity UPLC BEH T3 column (1.8 µm, 2.1 × 100 mm) maintained at 40 °C. The mobile phases consisted of solvent A (water with 0.01% formic acid) and solvent B (acetonitrile). The flow rate was set to 0.30 mL/min, and the injection volume was 6 µL. Mass spectrometry was conducted in both positive and negative ESI modes with the following parameters: curtain gas at 35 psi, collision gas at 7 psi, ion spray voltage of ±4500 V, ion source temperature at 450 °C, and both ion source gases (Gas 1 and Gas 2) at 35 psi. The LODs were approximately 0.5–2 ng/g, and the LOQs were in the range of 1–5 ng/g, depending on the compound and matrix.


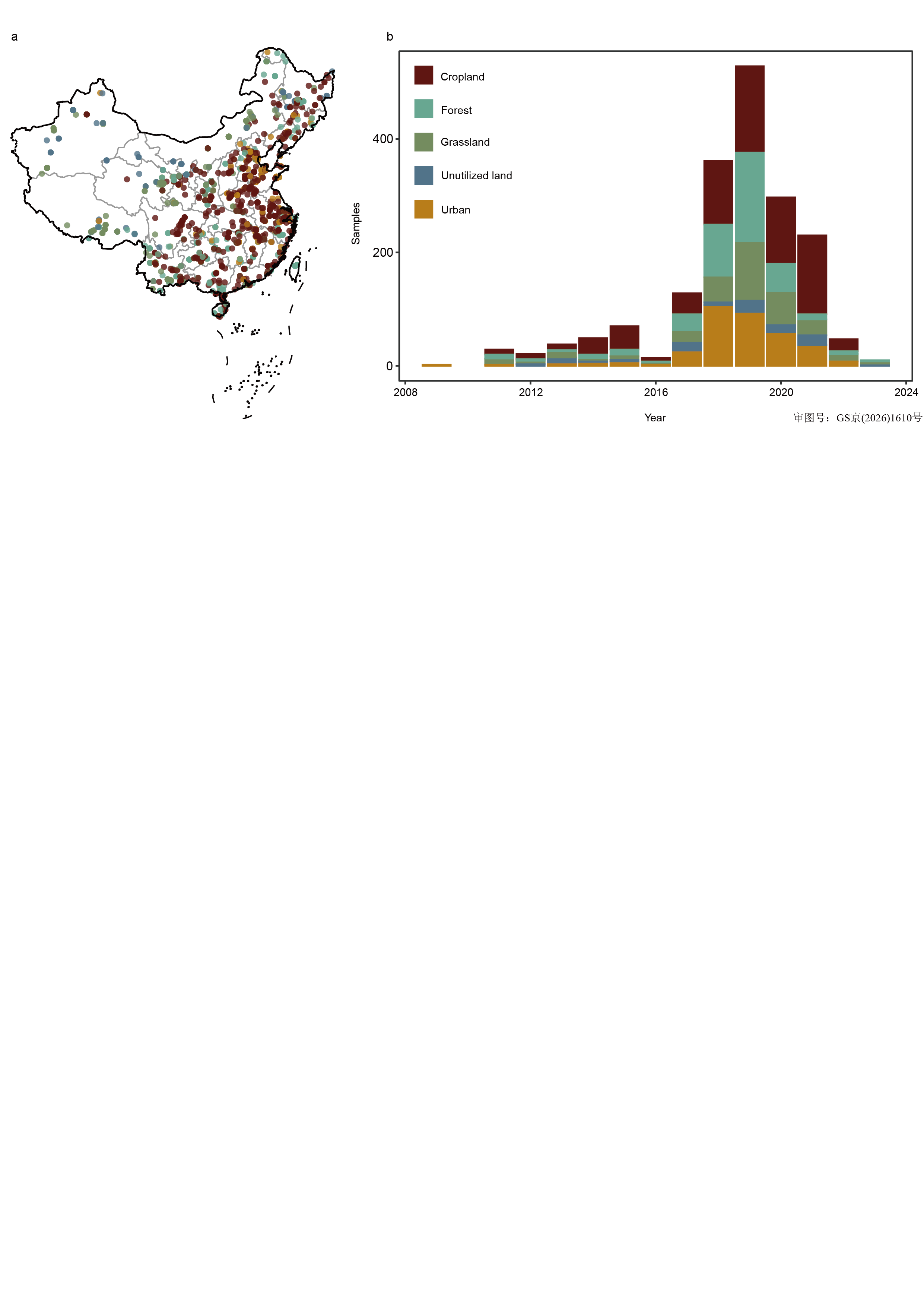


Fig. S1 The distribution of Chinese soil metagenomic data. (a) The distribution of public and in-house metagenomic data. (b) Distribution of sampling year and land use types.


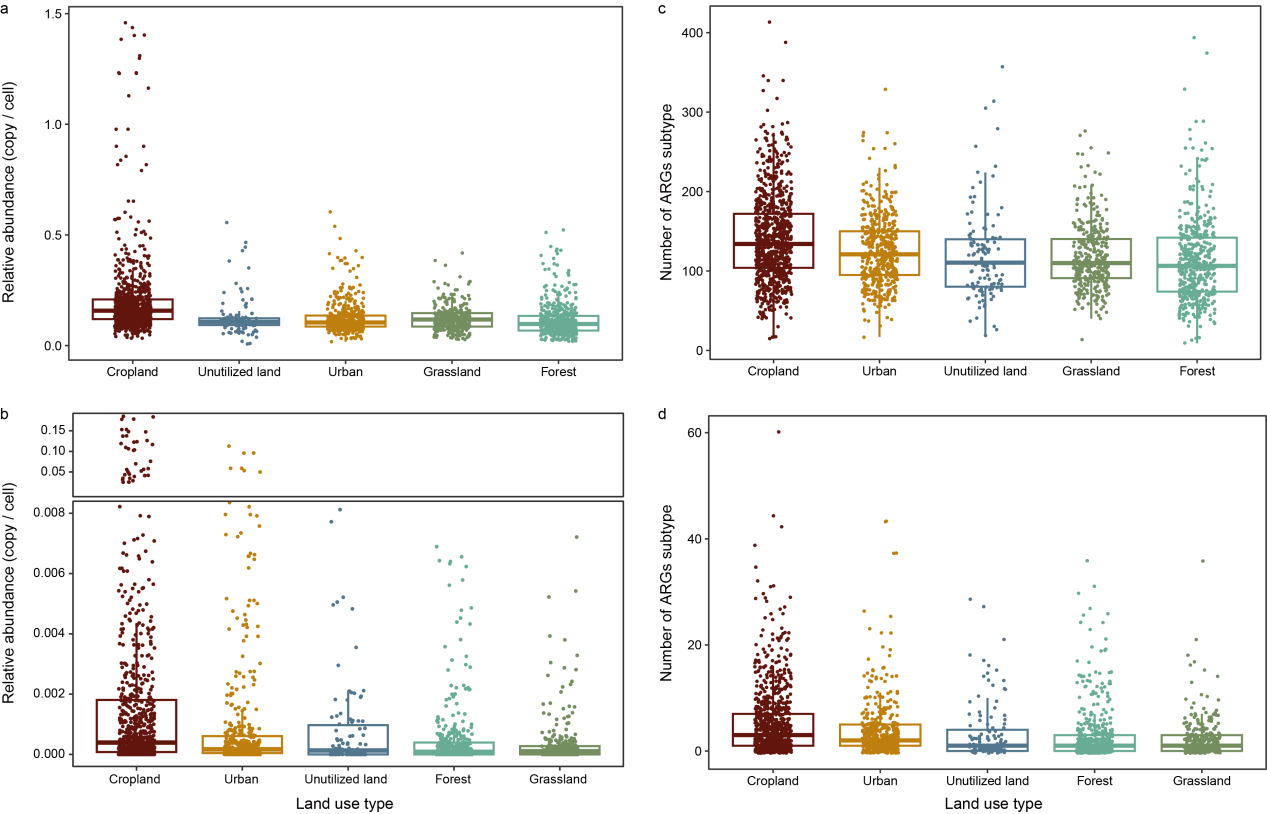


Fig. S2 The relative abundance and diversity of total ARGs and RankI ARGs in different land use type. a) The relative abundance of total ARGs. In the boxplots of panels, hinges indicate the 25th, 50th, and 75th percentiles, whiskers indicate 1.5× interquartile ranges (cropland: 867, Forest: 454, Grassland: 336, Unutilized land: 118, and Urban: 467). b) The relative abundance of Rank I ARGs. In the boxplots of panels, hinges indicate the 25th, 50th, and 75th percentiles, whiskers indicate 1.5× interquartile ranges (cropland: 867, Forest: 454, Grassland: 336, Unutilized land: 118, and Urban: 467). c) The diversity of total ARGs. In the boxplots of panels, hinges indicate the 25th, 50th, and 75th percentiles, whiskers indicate 1.5× interquartile ranges (cropland: 867, Forest: 454, Grassland: 336, Unutilized land: 118, and Urban: 467). d) The diversity of total ARGs. In the boxplots of panels, hinges indicate the 25th, 50th, and 75th percentiles, whiskers indicate 1.5× interquartile ranges (cropland: 867, Forest: 454, Grassland: 336, Unutilized land: 118, and Urban: 467).


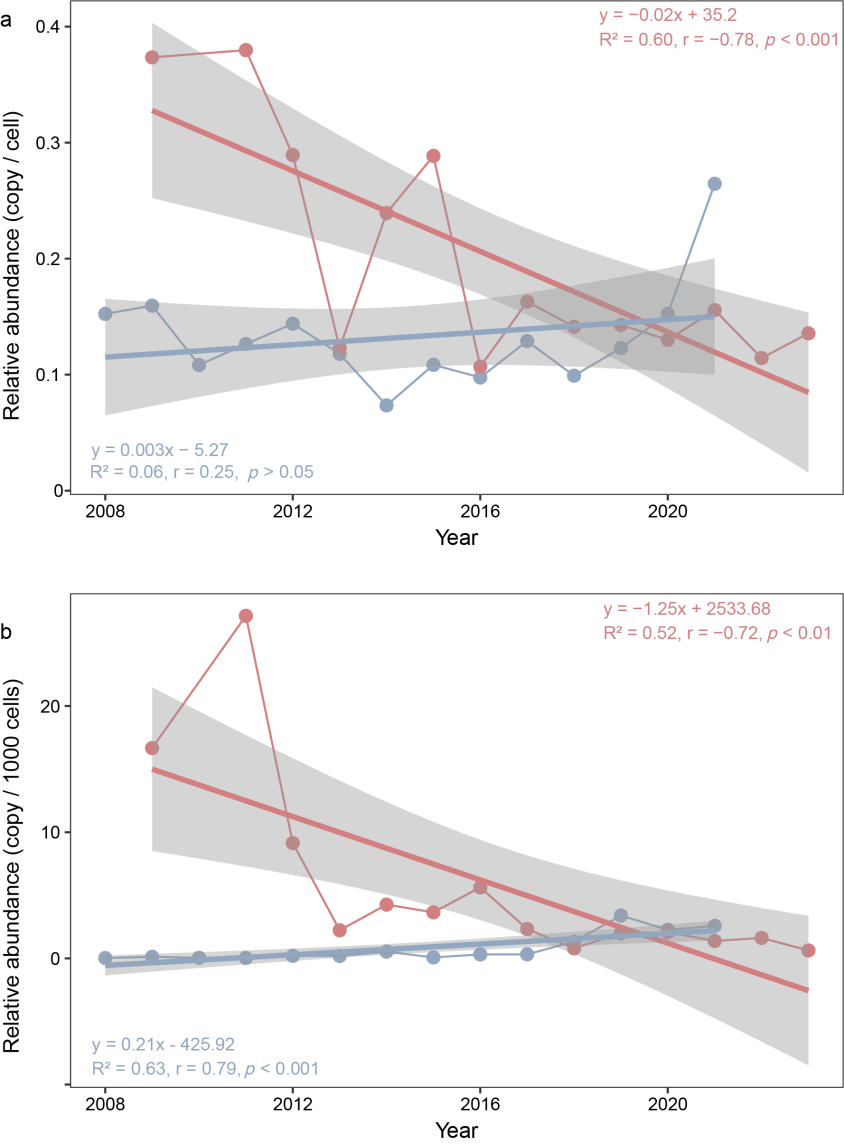


Fig. S3 Changes in the relative abundance of total ARGs and Rank I ARGs in Chinese and global soils over time. a) Total ARGs. b) Rank I ARGs.


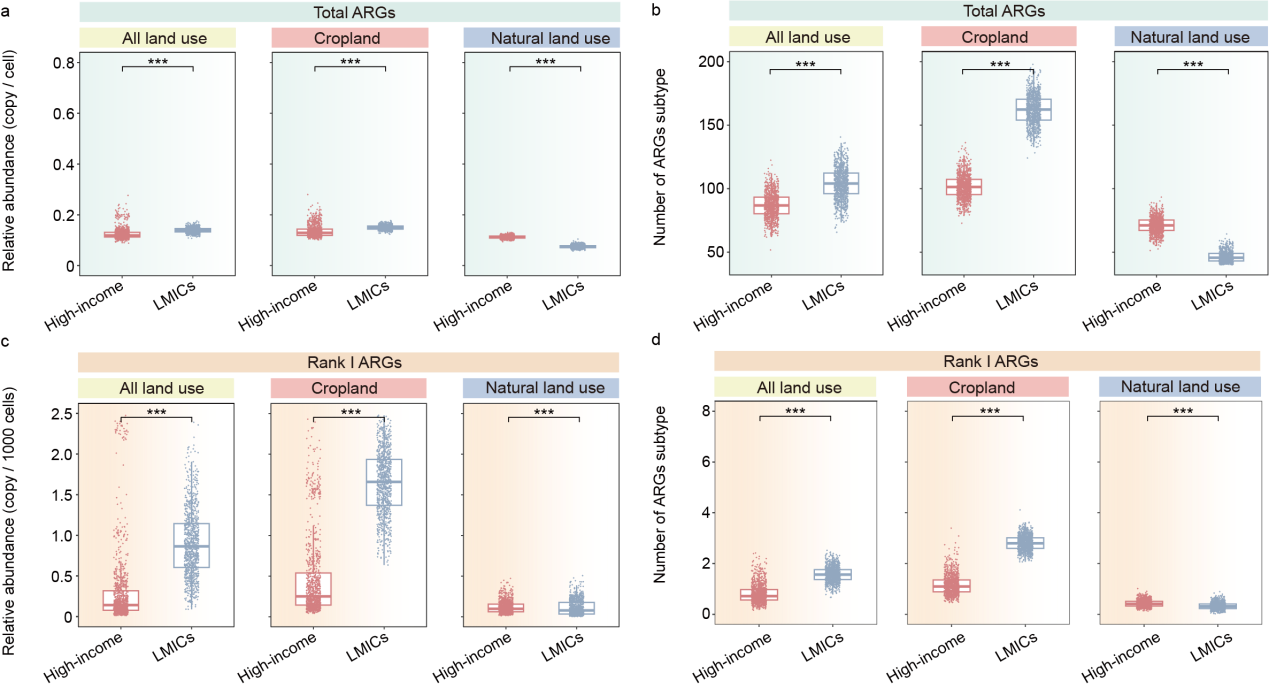


Fig. S4 Distribution of the relative abundance and risk of soil ARGs across countries with different income levels worldwide (excluding Chinese samples) according World Bank. a) The relative abundance of total ARGs. b) The relative abundance of Rank I ARGs. c) The diversity of total ARGs. d) The diversity of Rank I ARGs. The relative abundance were calculated across the three land use groups, including total land use type, cropland, and natural land use type. Total land use type included all the land use. The grassland, forest, and unutilized land were classified into natural land use type. In the comparison of total land use types, 999 rounds of sampling were performed based on the stable sample size in the stable land use types (cropland: 74, other landuse type: 74) for the calculation of both total ARGs and Rank I ARGs. In the boxplots of panels, hinges indicate the 25th, 50th, and 75th percentiles, whiskers indicate 1.5 × interquartile ranges, and dots indicate values of individual samples. Significant comparisons (two-sided *t*-test) between different periods are indicated by ***, *p* < 0.001.


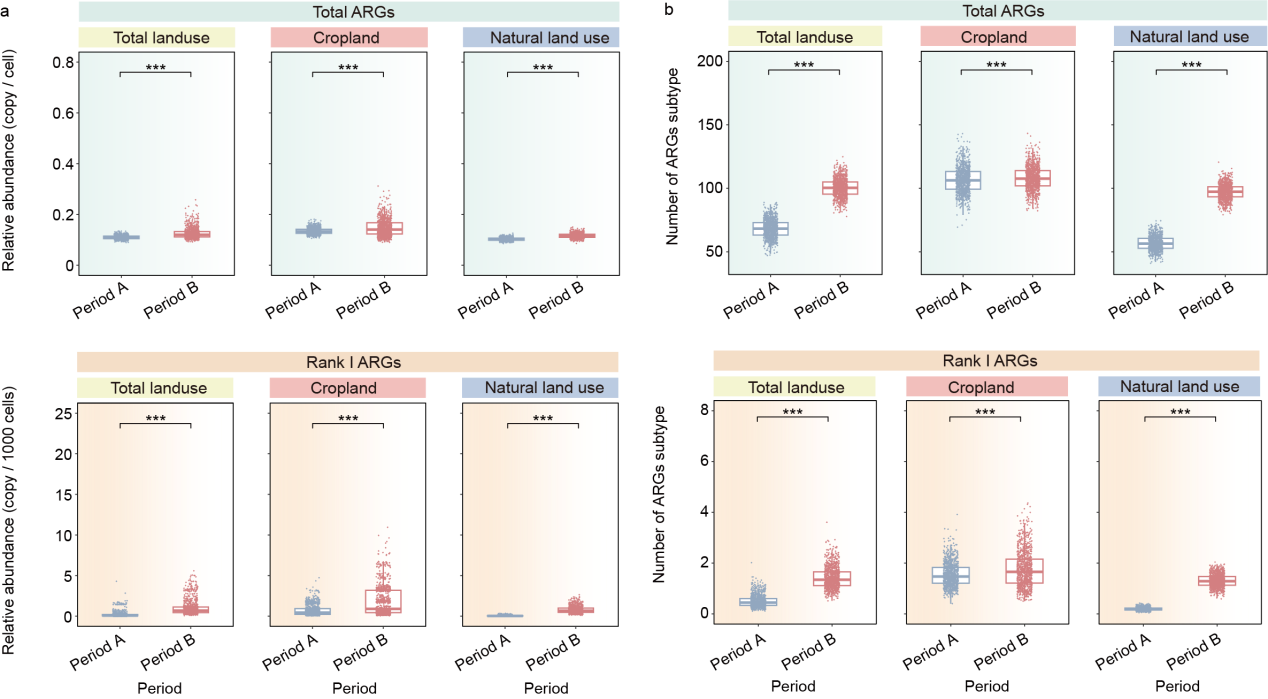


Fig. S5 Temporal changes in the relative abundance and risk of soil ARGs in global soils (excluding Chinese samples). a) Temporal changes in the relative abundance of total ARGs and Rank I ARGs in global soils (excluding Chinese samples). The samples were divided into two periods based on time, including period A (before 2015, 2008 - 2015) and period B (after 2016, 2016 - 2022). Temporal trends were calculated across the three land use groups, including total land use type, cropland, and natural land use type. Total land use type included all land uses. The grassland, forest, and unutilized land were classified into natural land use type. In the comparison of total land use types, 999 rounds of sampling were performed based on the stable sample size in the stable land use types (cropland: 90, forest: 30, grassland: 30, unutilized land: 10, urban: 10) for the calculation of both total ARGs and Rank I ARGs. In the comparison of cropland and natural land use types, 999 rounds of sampling were performed based on the stable sample size (cropland: 70, natural land use type: 70). In the boxplots of panels, hinges indicate the 25th, 50th, and 75th percentiles, whiskers indicate 1.5 × interquartile ranges, and dots indicate values of individual samples. Significant comparisons (two-sided *t*-test) between different periods are indicated by ***, *p* < 0.001. b) Temporal changes in the diversity of total ARGs and Rank I ARGs in global soils (excluding Chinese samples). The samples were divided into two periods based on time, including period A (before 2015, 2008 - 2015) and period B (after 2016, 2016 - 2022). Temporal trends were calculated across the three land use groups, including total land use type, cropland, and natural land use type. Total land use type included all land use types. The grassland, forest, and unutilized land were combined into natural land use type. In the comparison of total land use type, 999 rounds of sampling were performed based on the stable sample size (cropland: 90, forest: 30, grassland: 30, unutilized land: 10, urban: 10) for the calculation of both total ARGs and Rank I ARGs. In the comparison of cropland and natural land use types, 999 rounds of sampling were performed based on the stable sample size (cropland: 70, natural land use type: 70). In the boxplots of panels, hinges indicate the 25th, 50th, and 75th percentiles, whiskers indicate 1.5 × interquartile ranges, and dots indicate values of individual samples. Significant comparisons (two-sided *t*-test) between different periods are indicated by ***, *p* < 0.001.


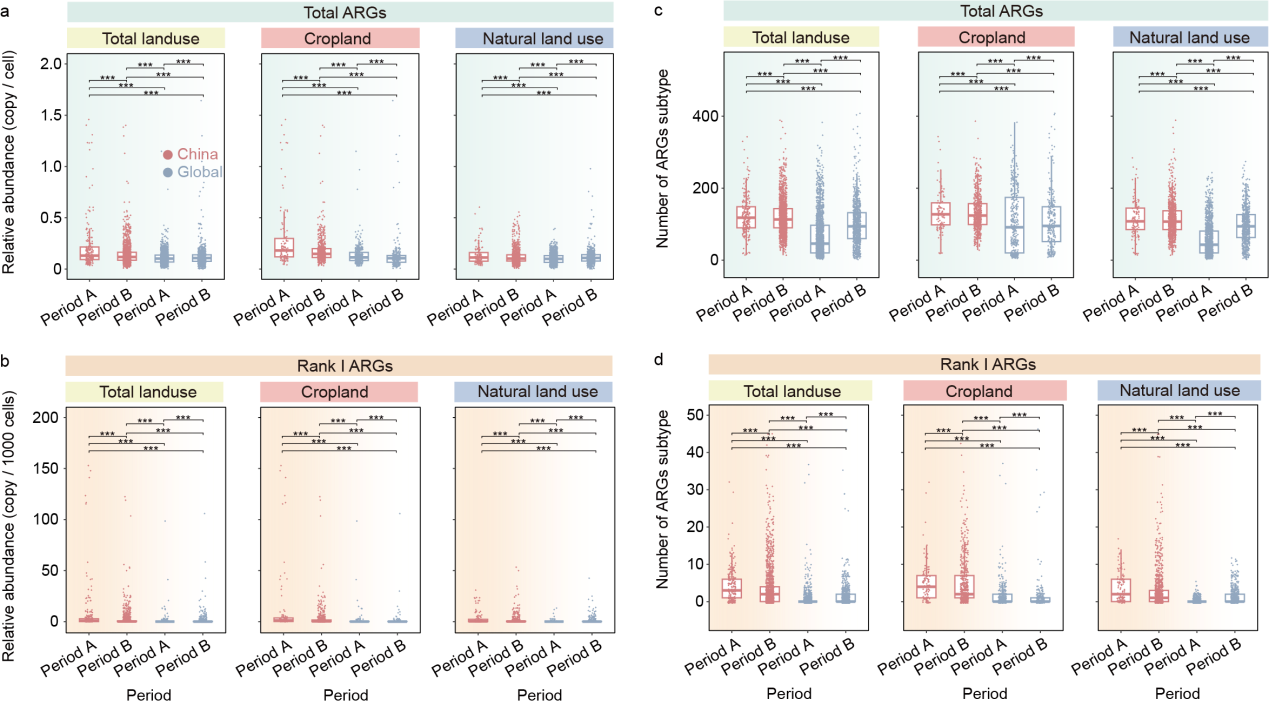


Fig. S6 Temporal changes in the relative abundance and risk of soil ARGs in China and global soil. a) Temporal changes in the relative abundance of total ARGs in China and global soil. b) Temporal changes in the relative abundance of Rank I ARGs in China and global soil. c) Temporal changes in the diversity of total ARGs in China and global soil. d) Temporal changes in the diversity of Rank I ARGs in China and global soil. Temporal trends were calculated across the three land use groups, including total land use type, cropland, and natural land use type. Total land use type included all the land use. The grassland, forest, and unutilized land were classified into natural land use type. Total: China Period A: 199 samples, China Period B: 1262 samples, Global Period A: 1663 samples, Global Period B: 893 samples. Cropland: China Period A: 104 samples, China Period B: 577 samples, Global Period A: 380 samples, Global Period B: 259. Natural land use type: China Period A: 95 samples, China Period B: 685 samples, Global Period A: 1232 samples, Global Period B: 625 samples.


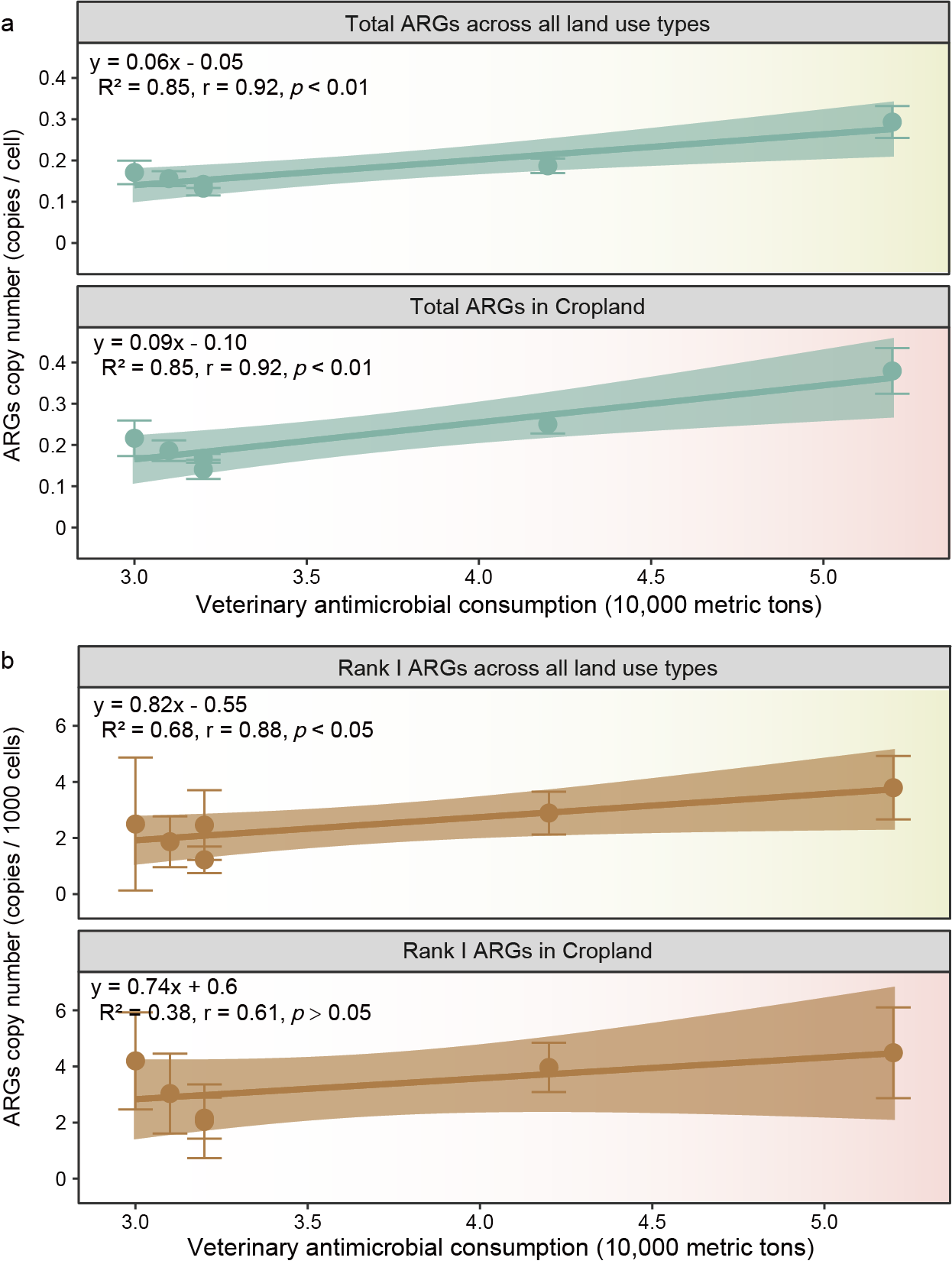


Fig. S7 The relationship between the **relative abundance** of total ARGs and Rank I ARGs in China and **Chinese veterinary antimicrobials consumption** (from 2015 - 2021). a) The relationship between the **relative abundance** of total ARGs in China and **Chinese veterinary antimicrobials consumption** (from 2015 - 2021). **2016 samples** were **excluded** due to the limits of samples size (14 samples). 999 rounds of sampling were performed based on the minimum sample size (cropland: 15, forest: 5, grassland: 2, unutilized land: 3, urban: 7) for the calculation of both total ARGs and Rank I ARGs. R^2^ was calculated from the linear model, and r was calculated from the Pearson correlation (liner regression). The correlation was statistically tested using both Pearson’s correlation test (two-sided) and a linear model (two-sided). Grey shading denotes the 95% confidence intervals. b) The relationship between the **relative abundance** of Rank I ARGs in China and **Chinese veterinary antimicrobials consumption** (from 2015 - 2021). **2016 samples** were **excluded** due to the limits of samples size (14 samples). 999 rounds of sampling were performed based on the minimum sample size (cropland: 15, forest: 5, grassland: 2, unutilized land: 3, urban: 7) for the calculation of both total ARGs and Rank I ARGs. R^2^ was calculated from the linear model, and r was calculated from the Pearson correlation (liner regression). The correlation was statistically tested using both Pearson’s correlation test (two-sided) and a linear model (two-sided). Grey shading denotes the 95% confidence intervals.


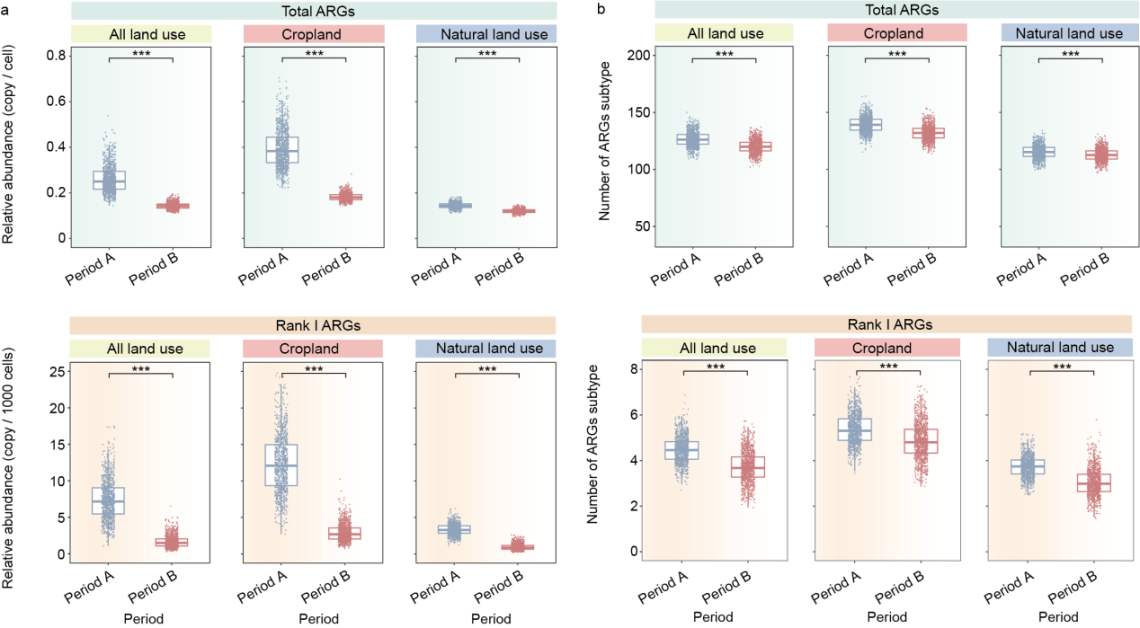


Fig. S8 Temporal changes in the relative abundance and risk of soil ARGs in China (excluding samples from 2016). a) Temporal changes in the relative abundance of total ARGs. b) Temporal changes in the relative abundance of Rank I ARGs. c) Temporal changes in the diversity of total ARGs. d) Temporal changes in the diversity of Rank I ARGs. Temporal trends were calculated across the three land use groups, including total land use type, cropland, and natural land use type. Total land use type included all the land use. The grassland, forest, and unutilized land were classified into natural land use type. In the comparison of total land use types, 999 rounds of sampling were performed based on the stable sample size in the stable land use **types** (cropland: 90, forest: 30, grassland: 30, unutilized land: 10, urban: 10) for the calculation of both total ARGs and Rank I ARGs. In the comparison of cropland and natural land use types, 999 rounds of sampling were performed based on the stable sample size (cropland: 70, natural land use type: 70). In the boxplots of panels, hinges indicate the 25th, 50th, and 75th percentiles, whiskers indicate 1.5 × interquartile ranges, and dots indicate values of individual samples. Significant comparisons (two-sided *t*-test) between different periods are indicated by ***, *p* < 0.001.


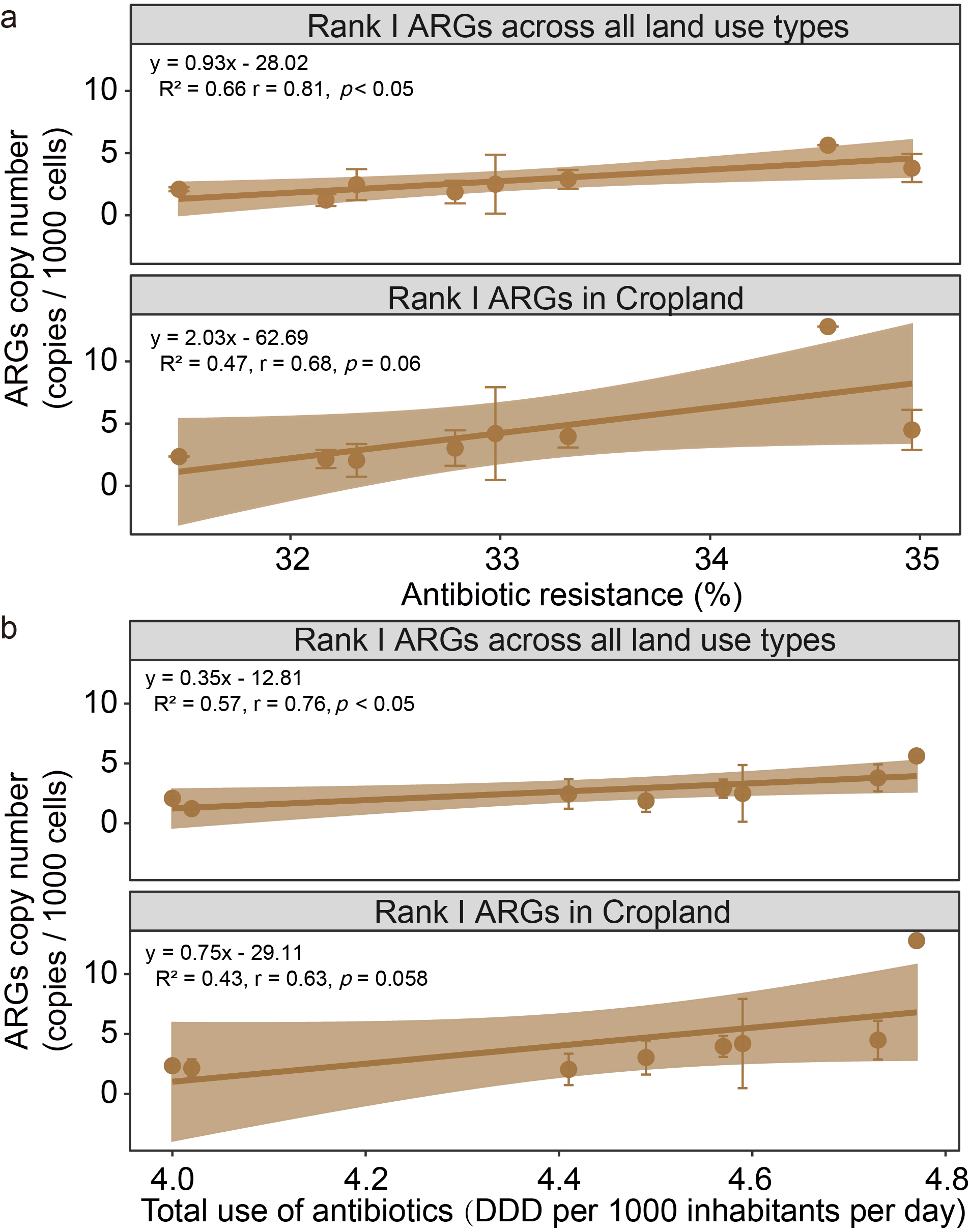


Fig. S9 The relationship between the relative abundance of ARGs and Rank I ARGs in China and Chinese clinical antibiotic resistance (from 2015 - 2022). a) The relationship between the relative abundance of ARGs and Rank I ARGs in China and Chinese clinical antibiotic resistance (from 2015 - 2022). 999 rounds of sampling were performed based on the minimum sample size (cropland: 15, forest: 5, grassland: 2, unutilized land: 3, urban: 7) for the calculation of both total ARGs and Rank I ARGs. R^2^ is calculated from the linear model, and r is calculated from the Pearson correlation (liner regression). The correlation was statistically tested using both Pearson’s correlation test (two-sided) and a linear model (two-sided). Grey shading denotes the 95% confidence intervals. Antibiotic resistance was calculated as the mean value based on the 13 collected pathogens. b) The relationship between the relative abundance of ARGs and Rank I ARGs in China and Chinese antibiotic consumption intensity (from 2015 - 2022). 999 rounds of sampling were performed based on the minimum sample size (cropland: 15, forest: 5, grassland: 2, unutilized land: 3, urban: 7) for the calculation of both total ARGs and Rank I ARGs. R^2^ is calculated from the linear model, and r is calculated from the Pearson correlation (liner regression). The correlation was statistically tested using both Pearson’s correlation test (two-sided) and a linear model (two-sided). Grey shading denotes the 95% confidence intervals.


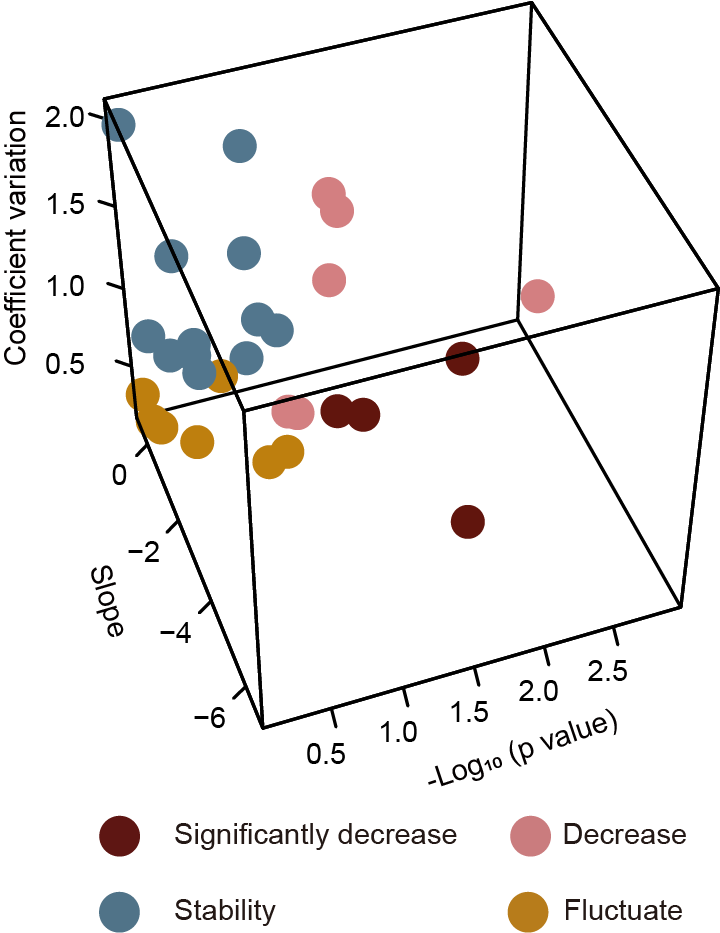


Fig. S10 Changes in soil ARG types in China from 2015 to 2022 (2016 excluded), categorized as significant increase (slope > 0.1, p ≤ 0.05), increase (slope > 0.1, 0.05 < p ≤ 0.1), significantly decreased (slope < -0.1, p ≤ 0.05), decreased (slope < -0.1, 0.05 < p ≤ 0.1), stabilized (p > 0.1, CV < 0.5), and fluctuated (p > 0.1, CV ≥ 0.5).


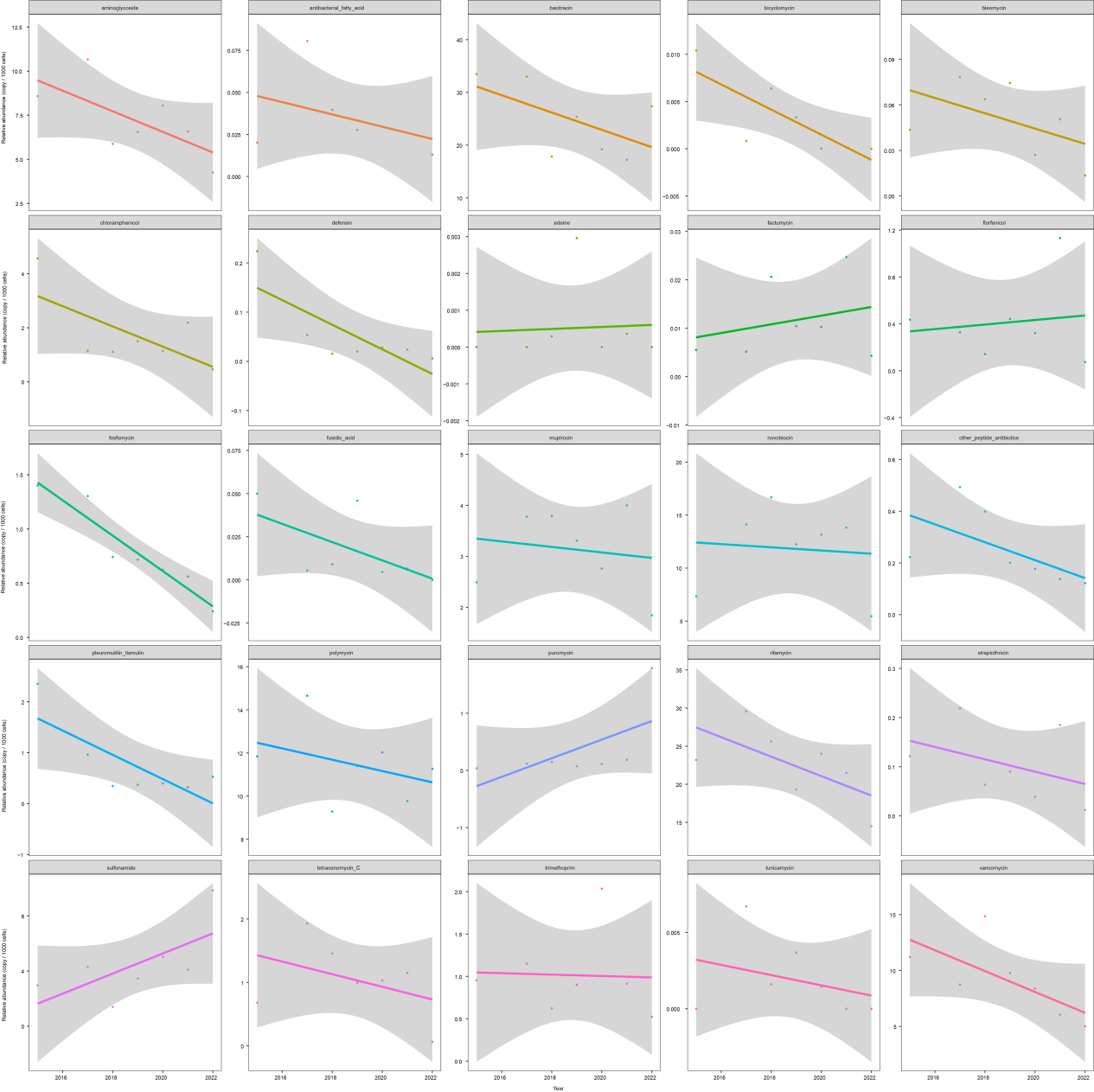


Fig. S11 Patterns of change over time for the remaining 25 ARGs type.


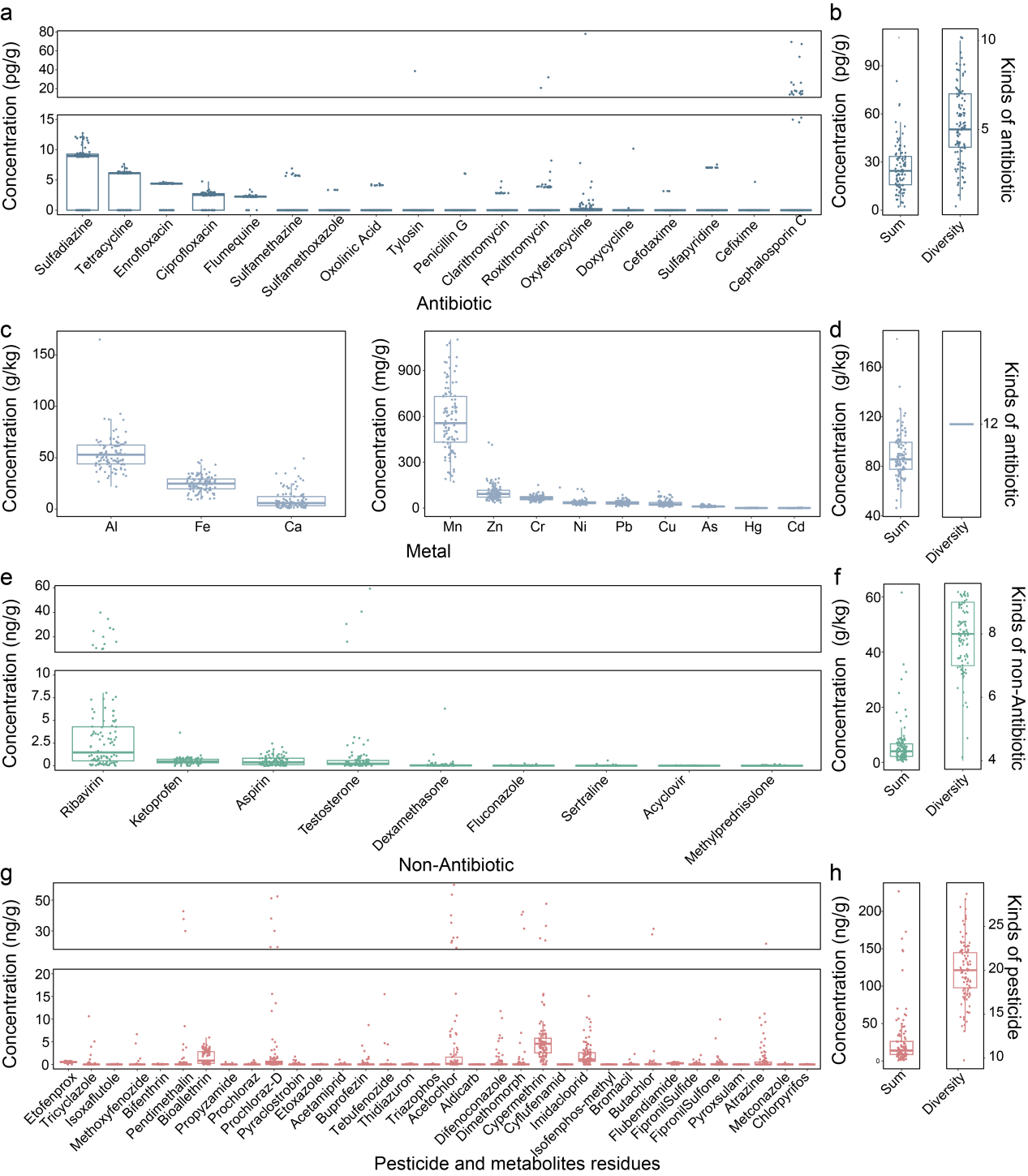


Fig. S12 Chemical contaminants in cropland in 2021. a) The concentration of antibiotic. b) the total concentration and kinds of antibiotic. c) The concentration of metal. d) the total concentration and kinds of metal. e) The concentration of non-antibiotic drugs. f) the total concentration and kinds of non-antibiotic drugs. g) The concentration of pesticide and metabolites residues. h) the total concentration and kinds of pesticide and metabolites residues.


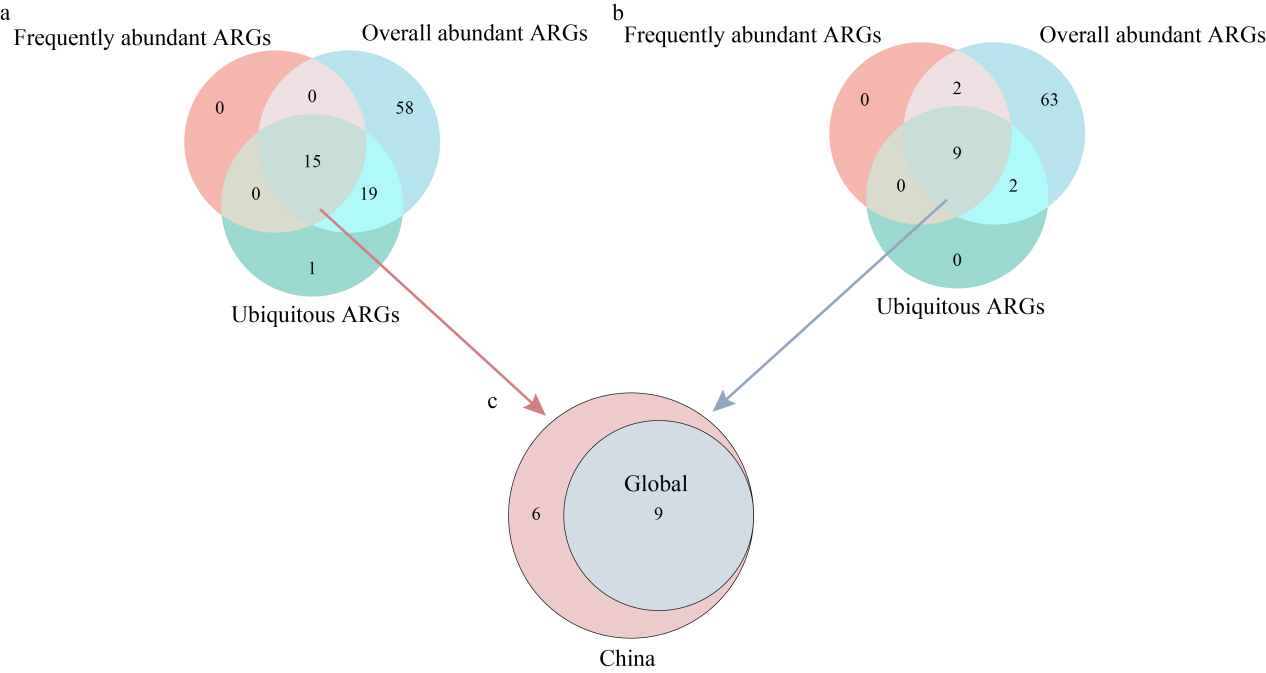


Fig. S13 The selection of core ARGs in Chinese and global soils. a) The core ARGs in Chinese soil. b) The core ARGs in global soils. c) The difference of core ARGs in Chinese and global soils.


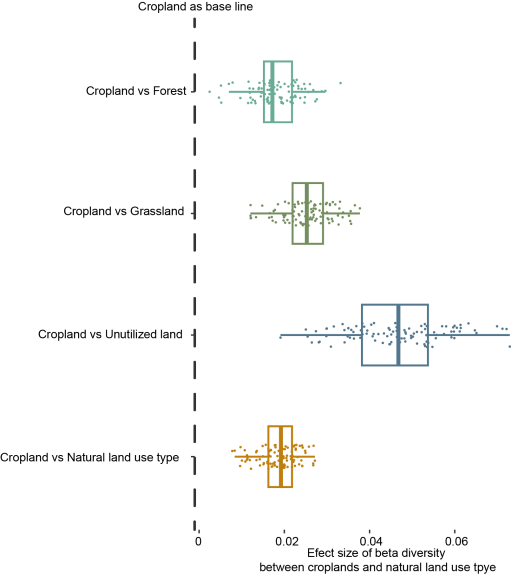


Fig. S14 Effect sizes of natural ecosystems' impacts on β-diversity, with croplands as the baseline. Each point represents the difference in β-diversity between a given land use type and cropland. Nature land use type includes forest, grassland, and unutilized land. To ensure the reliability of the results, we balanced the number of samples based on their source (China vs. global) and habitat (cropland vs. other) and repeated the calculation 100 times. Specifically, for the comparison between cropland and forest, we had 454 samples from both China and global cropland, and 454 samples from both China and global forest. For cropland vs. grassland, we had 336 samples from both China and global cropland, and 336 samples from both China and global grassland. For cropland vs. unutilized land, we had 118 samples from both China and global cropland, and 118 samples from both China and global unutilized land. For cropland vs. natural land type, we had 639 samples from both China and global cropland, and 639 samples from both China and global natural land type. In the boxplots of panels, hinges indicate the 25th, 50th, and 75th percentiles, whiskers indicate 1.5× interquartile ranges, and dots indicate a single β-diversity calculation (100 in total).


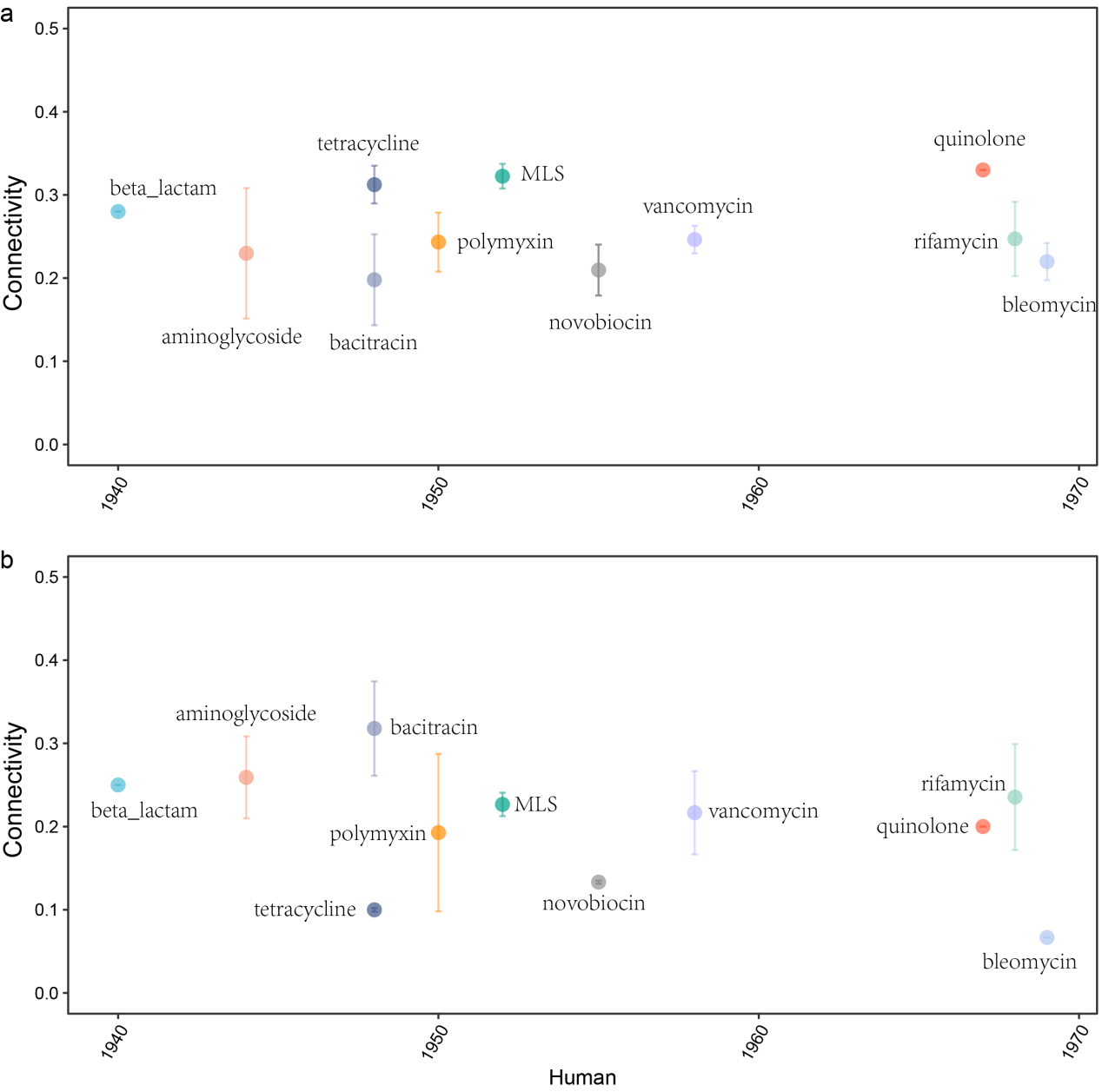


Fig. S15 The relationship between the connectivity of ARGs type and the year of antibiotic introduction. a) Cropland. The error bars were derived from the subtypes belonging to this type. b) Land use type. The error bars were derived from the subtypes belonging to this type.

Table S1 Adonis analysis of ARGs composition

| **Group pairs** | **Sum.sq** | **R^2^** | **F** | **Pr(>F)** | **Significant** |
| --- | --- | --- | --- | --- | --- |
| **Total ARGs** | 19.772 | 56.431 | 0.09059 | 0.001 | **Yes** |
| **RankI ARGs** | 7.96 | 4.2848 | 0.1994 | 0.001 | **Yes** |

Table S2 Significance test for differences between various land use type (total ARGs)

| **Group pairs** | **Sum.sq** | **F** | **R2** | **Pr(>F)** | **Significant** |
| --- | --- | --- | --- | --- | --- |
| cropland vs Forest | 9.7530 | 107.5556 | 0.0752 | 0.001 | Yes |
| cropland vs Grassland | 9.6470 | 112.7023 | 0.0844 | 0.001 | Yes |
| cropland vs Unutilized land | 4.2249 | 46.0006 | 0.0443 | 0.001 | Yes |
| cropland vs Urban | 9.4275 | 110.9497 | 0.076 | 0.001 | Yes |
| Forest vs Grassland | 1.4657 | 16.71494 | 0.0202 | 0.001 | Yes |
| Forest vs Unutilized land | 0.7773 | 7.8316 | 0.0133 | 0.001 | Yes |
| Forest vs Urban | 0.54233 | 6.2679 | 0.0067 | 0.001 | Yes |
| Grassland vs Unutilized land | 0.3396 | 3.8540 | 0.0080 | 0.003 | Yes |
| Grassland vs Urban | 0.8263 | 10.5320 | 0.0126 | 0.001 | Yes |
| Unutilized land vs Urban | 0.6791 | 7.8829 | 0.0131 | 0.001 | Yes |

Table S3 Significance test for differences between various land use type

(Rank I ARGs)

| **Group pairs** | **Sum.sq** | **F** | **R2** | **Pr(>F)** | **Significant** |
| --- | --- | --- | --- | --- | --- |
| Forest vs cropland | 1.9508 | 4.1704 | 0.0039 | 0.001 | Yes |
| Forest vs Urban | 0.8659 | 1.8533 | 0.0026 | 0.001 | Yes |
| Forest vs Grassland | 2.0902 | 4.5086 | 0.0080 | 0.001 | Yes |
| Forest vs Unutilized land | 1.3799 | 2.9500 | 0.0072 | 0.001 | Yes |
| cropland vs Urban | 1.6970 | 3.6448 | 0.0033 | 0.001 | Yes |
| cropland vs Grassland | 3.9120 | 8.4454 | 0.0087 | 0.001 | Yes |
| cropland vs Unutilized land | 1.3757 | 2.9570 | 0.0036 | 0.001 | Yes |
| Urban vs Grassland | 2.3968 | 5.2106 | 0.0085 | 0.001 | Yes |
| Urban vs Unutilized land | 1.3594 | 2.9391 | 0.0063 | 0.001 | Yes |
| Grassland vs Unutilized land | 1.1371 | 2.5067 | 0.0080 | 0.001 | Yes |

**REFERENCES**

1. Bolger AM, Lohse M, Usadel B. Trimmomatic: A Flexible Trimmer for Illumina Sequence Data. *Bioinformatics* 2014; **30**: 2114-20.

2. Yin X, Zheng X, Li L *et al.* Args-Oap V3.0: Antibiotic-Resistance Gene Database Curation and Analysis Pipeline Optimization. *Engineering* 2023; **27**: 234-41.

3. Yin X, Chen X, Jiang X *et al.* Toward a Universal Unit for Quantification of Antibiotic Resistance Genes in Environmental Samples. *Environ Sci Technol* 2023; **57**: 9713-21.

4. Hernando-Amado S, Coquet TM, Baquero F *et al.* Defining and Combating Antibiotic Resistance From One Health and Global Health Perspectives. *Nat Microbiol* 2019; **4**: 1432-42.

5. Li D, Liu C, Luo R *et al.* Megahit: An Ultra-Fast Single-Node Solution for Large and Complex Metagenomics Assembly Via Succinct De Bruijn Graph. *Bioinformatics* 2015; **31**: 1674-6.

6. Hyatt D, Chen G, Locascio PF *et al.* Prodigal: Prokaryotic Gene Recognition and Translation Initiation Site Identification. *Bmc Bioinformatics* 2010; **11**.

7. Parnanen K, Karkman A, Hultman J *et al.* Maternal Gut and Breast Milk Microbiota Affect Infant Gut Antibiotic Resistome and Mobile Genetic Elements. *Nat Commun* 2018; **9**.

8. Camargo AP, Roux S, Schulz F *et al.* Identification of Mobile Genetic Elements with Genomad. *Nat Biotechnol* 2024; **42**.

9. Parks DH, Chuvochina M, Waite DW *et al.* A Standardized Bacterial Taxonomy Based On Genome Phylogeny Substantially Revises the Tree of Life. *Nat Biotechnol* 2018; **36**: 996.

10. Zhao Y, Li L, Huang Y *et al.* Global Soil Antibiotic Resistance Genes are Associated with Increasing Risk and Connectivity to Human Resistome. *Nat Commun* 2025; **16**: 7141.

11. Pal C, Bengtsson-Palme J, Rensing C *et al.* Bacmet: Antibacterial Biocide and Metal Resistance Genes Database. *Nucleic Acids Res* 2014; **42**: D737-43.

12. Li H, Liu Z, Hu B *et al.* Distribution of Tetracyclines and Sulfonamides Resistance Genes Around a Smallholder Pig Farm: Modeling and Attribution. *Sci Total Environ* 2024; **954**.

13. Yu Z, Liu Z, Sun L *et al.* Mobile Genetic Elements Mediate the Cross-Media Transmission of Antibiotic Resistance Genes From Pig Farms and their Risks. *Sci Total Environ* 2024; **926**.
